# Synthesis and Anti-Hepatoma Activities of U12 Derivatives Arresting G0/G1 Phase and Inducing Apoptosis by PI3K/AKT/mTOR Pathway

**DOI:** 10.3390/ph15010107

**Published:** 2022-01-17

**Authors:** Renjing Yang, Chunchun Du, Ting Cao, Guanghui Wang, Xin Jiang, Jun Gao, Ting Lin, Cuiling Sun, Rong Ding, Wenjing Tian, Haifeng Chen

**Affiliations:** Fujian Provincial Key Laboratory of Innovative Drug Target, School of Pharmaceutical Sciences, Xiamen University, Xiamen 361005, China; fzmu__yrj@163.com (R.Y.); DuChunchun97@163.com (C.D.); caoting_001@163.com (T.C.); guanghui@xmu.edu.cn (G.W.); 32320130154303@stu.xmu.edu.cn (X.J.); 33920192204365@stu.xmu.edu.cn (J.G.); linting@xmu.edu.cn (T.L.); cuiling@xmu.edu.cn (C.S.); dingrong@xmu.edu.cn (R.D.)

**Keywords:** U12 derivatives, U12a, anti-hepatoma activity

## Abstract

Ursodeoxycholic acid (UDCA) is a first-line clinical drug for the treatment of liver diseases. U12, a derivative of UDCA, showed effective anti-hepatoma activities in previous works. However, the low polarity and large doses limited the druglikeness of U12. In this study, the structural modification and optimization of U12 were further investigated and twelve U12 derivatives were synthesized by substitution, esterification and amidation reactions. The evaluation of the cytotoxicity of synthetic derivatives against hepatoma cell lines (HepG2) indicated that U12-I, U12a-d and U12h showed more effective cytotoxic effects on the growth of HepG2 cells than U12, and the preliminary structure–activity relationship was discussed. Among them, U12a exhibited the most potent anti-hepatocellular carcinoma activity. Mechanism studies indicated that U12a inhibited HepG2 cell proliferation by arresting the G0/G1 phase, and suppressed the activation of the PI3K/AKT/mTOR pathway. Further studies showed that U12a induced HepG2 cells apoptosis through activating the caspase signaling pathway. Furthermore, U12a evidently inhibits the growth of HepG2-derived tumor xenografts in vivo without observable adverse effects. Thus, U12a might be considered as a promising candidate for the treatment of hepatocellular carcinoma.

## 1. Introduction

Hepatocellular carcinoma (HCC), one of the most lethal cancers, is highly metastatic and invasive. The International Agency for Research on Cancer (IARC) released that more than 910,000 new cases of HCC happened, and approximately 830,000 people died of liver cancer, worldwide in 2020 [1]. The mortality rate of liver cancer ranks the third in the world. Cirrhosis, the hepatitis B and C virus infections, excessive alcohol consumption and obesity are the main causes of HCC. Recurrence and metastasis result in an unoptimistic prognosis in HCC [2,3]. Most HCC patients are diagnosed at the advanced stages with limited therapies. Sorafenib is the only FDA-approved first-line systemic therapy for advanced HCC, while regorafenib and nivolumab are the second-line therapies for HCC after sorafenib failure [4,5,6]. Therefore, to find a new efficient chemotherapeutic agent for HCC treatment is of great urgency.

Ursodeoxycholic acid (UDCA), a hydrophilic bile acid, has been widely used clinically as a safe medical therapy for liver diseases, especially for the primary biliary cirrhosis (PBC) and cholestatic liver disease [7,8]. In recent years, studies have shown that UDCA exhibits protective effects on hepatocytes and non-hepatic cells. Moreover, it could induce apoptosis of human hepatocarcinoma cell lines (HepG2), as well as block the occurrence of colon cancer, which was evaluated in clinical phase III trials for the prevention of colorectal adenoma recurrence [9,10,11,12]. Besides, UDCA is known to prevent gastrointestinal disorders of patients with stomach, colon, lung, breast or liver cancers [13]. Although UDCA has outstanding curative effects on hepatobiliary diseases, the patient needs to take large dosages (500–1000 mg/d) to reach the effective dose. Therefore, more and more researchers choose UDCA as the lead compound to modify its structure, in order to obtain the synthetic derivatives with more potent therapeutic effects. NorUDCA (24-norursodeoxycholic acid), the side chain-shortened C23-homologue of UDCA, showed remarkable therapeutic effects in cholestatic Mdr2 (Abcb4) knockout mice [14,15,16]. Some researches synthesized several UDCA derivatives with potent anti-tumor effects against numerous human cancer cells, which are conjugates of UDCA with amino acids by an N-acyl bond [17,18]. For instance, HS-1183, a conjugate of UDCA with an L-phenylalanine benzyl ester, was reported to exert anti-tumor effects by inducing the apoptosis of human breast and prostate cancer cell lines through a p53-independent pathway, and preventing the death of human cervical carcinoma cells via nuclear translocation of NF-κB and activation of the JNK pathway, while it was easily to be cleaved by intestinal and hepatic enzymes [19,20,21]. Another effective way to improve the cytotoxic activity against cancer cells was to introduce nitrogen heterocycles, especially for the piperazine and cinnamylpiperazinyl group [22,23].

**Figure 1:**
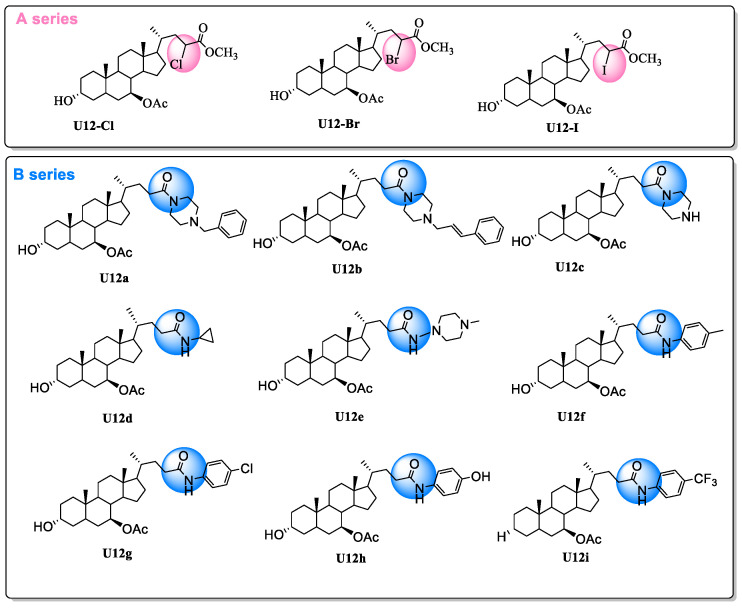
U12 derivatives of two series (**A**,**B**).

In our previously research, U12, a UDCA derivative, was found to show anti-hepatoma activities via mTOR/S6K1, cyclinD1/CDK2/4 and caspase-dependent apoptotic signaling pathways in hepatocellular carcinoma cells. Furthermore, the effects of U12 in vivo were investigated by a tumor xenograft mouse model, which confirmed that U12 exerted more potent anti-tumor activity than UDCA, and had less severe side effects than fluorouracil (5-Fu) [24]. However, the low polarity and large doses limited the druglikeness of U12. Thus, it is of great urgency to find more active UDCA derivatives with low toxicity. In the present study, UDCA was used as the raw material to further investigate the structural modification and optimization of U12, in order to find the derivatives endowed with more potent pharmacological profiles and clarify the structure–activity relationships. As we know, the introduction of halogen atoms has a positive effect on the druglikeness of the compound [25]. Besides, the introduction of nitrogen heterocycles greatly improved the anti-tumor activities of bile acid derivatives in the previous studies [22,23]. In this study, twelve U12 derivatives of two series (A and B) were synthesized by substitution, esterification and amidation reactions (Figure 1). The A series were halogen-substituted derivatives, and the B series were conjugates of UDCA with nitrogen heterocycles, cyclopropane, or substituted benzene rings by an N-acetyl moiety. The synthesis, cytotoxic activities, pro-apoptotic mechanism and structure–activity relationship were investigated.

## 2. Results and Discussion

### 2.1. Chemistry

The starting compound, UDCA (3α,7β-dihydroxycholanic acid), was acetylated with acetic anhydride in ethylacetate, taking 4-dimethylaminopyridine (DMAP) as a catalyst, to give the acetate U-I. Then U-I was reacted with chlorine sulfide (SOCl_2_) and further mixed with Br_2_ and pyridine to afford the halogen substituted mixture. Furthermore, the mixture was dissolved in the CH_2_Cl_2_ and further dripped into the cooling methanol to afford the Cl- and Br- substituted derivatives (U12-Br, U12-Cl). The U12-Br was reacted with NaI and Bu_4_N^+^I^−^ in acetone, and extracted with ethyl acetate to afford the U12-I.

**Scheme 1:**
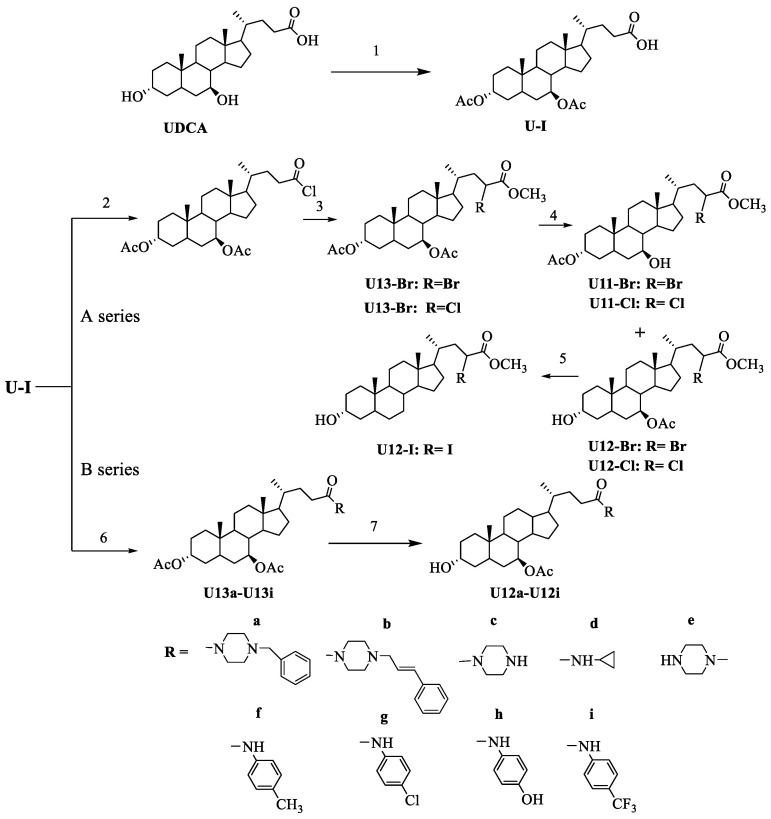
Synthesis of U12 derivatives. Reagents and conditions: (1) Ac_2_O, 4-DMAP, Py, r.t., 10 h; (2) SOCl_2_, 40 °C, 1 h; (3) Br_2_, Py, SOCl_2_, 70 °C, 6 h; (4) CH_2_Cl_2_, MeOH, 4 °C; (5) NaI, Bu_4_N^+^I^−^, acetone, r.t., 6 h; (6) HOBt, EDC·HCl, DMF, 4 °C, 1 h; a-i, 4℃, 24 h; (7) NaOH, MeOH, THF, r.t., 1 h.

The intermediate U-I reacted with 1-hydroxy benzotriazole (HOBt) and 1-(3-di methylaminopropyl-3-ethylcarbodiimide hydrochloride) (EDC·HCl) in the presence of the catalyst N,N-dimethylformamide (DMF), under the protection of N_2_ in an ice bath, and then reacted with the substituted piperazines [benzylpiperazine (a), trans-1-cinnamy lpiperazine (b), piperazine(c), cyclopropylamine(d),1-amino- 4-methylpiperazine (e), 4-Aminotoluene (f), p-Chloroaniline (g), 4-aminophenol (h), 4-Aminobenzotrifluoride (i)] to obtain the carboxamide derivatives of U13 (U13a-U13i). As U13 is the C-3 acetylated derivative of U12, the acetyl group at C-3 in the amides U13a-U13i were hydrolyzed to the hydroxyl group saponifying by methanolic potassium hydroxide to give the corresponding deacetylated amides U12a-U12i (Scheme 1). 

The structures of these synthesized compounds were characterized by the HR-MS, ^1^H NMR and ^13^C NMR spectra. For the A series of halogen-substituted derivatives of U12, Br-U12 was chosen for the descriptive example. The HR-ESI-MS spectrum of Br-U12 indicated the existences of one bromine atom with the relative isotope peak intensities of 1:1 ([M+Na]^+^: [M+Na+2]^+^). Compared with the 1D NMR data of U12, the chemical shifts of C-20, C-22 and C-23 in Br-U12 were downshifts to *δ* 34.1, 40.7 and 45.7, respectively, which indicated that the H-23 at the α-position of the carboxyl group was substituted by the Br atom. For the B series of U12 conjugates, U12a was chosen for the example of structure elucidation. Except for the expected signals for the steroid core of U12, the additional signals of the benzylpiperazine group [*δ*_C_ 133.7(C1, Ph), 129.3 × 2(C3 and C5, Ph), 128.5(C2 and C6, Ph), 127.5(C4, Ph), 63.0(C-Ph), 53.3 (CH_2_-, piperazinyl), 52.9(CH_2_-, piperazinyl)] in the ^13^C NMR spectrum indicated the formation of piperazinylsteroid carboxamide. In addition, the HR-ESI-MS analysis of U12a gives the molecular formula of C_37_H_56_O_4_N_2_, which further confirmed the formation of U12a.

### 2.2. Biological Activities

#### 2.2.1. Cytotoxic Effects of the U12 Derivatives on HepG2 Cells

**Figure 2:**
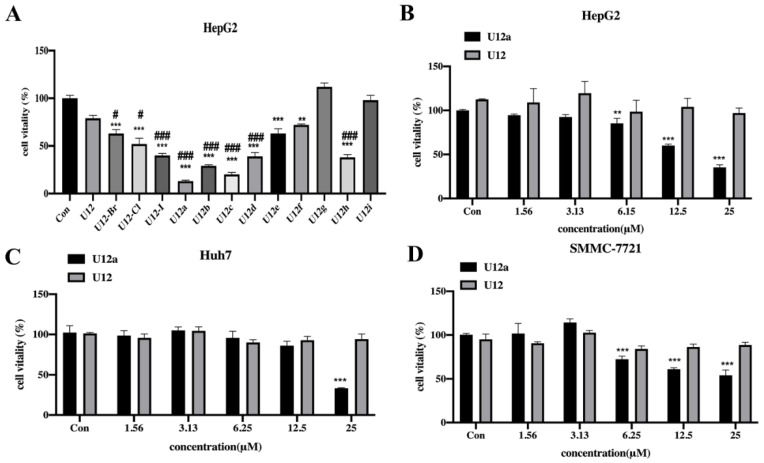
Cytotoxic effects of the U12 derivatives on HepG2 cells. (**A**) Cell viability of HepG2 cells after 24 h exposure with U12 derivatives (25 μM) by MTT assay. (**B**–**D**) Effects of U12a and U12 on viability of HepG2, Huh7, SMMC-7721 cells by MTT assay. (HepG2 cells were treated with different concentrations of U12a or U12 for 24 h, stained with MTT, and the OD value was measured at a wavelength of 490 nm.) All data represent the mean ± SD of three independent experiments. ** *p* < 0.01, *** *p* < 0.001 vs. control group, # *p* < 0.05, ### *p* < 0.001 vs. U12 group.

The cytotoxic effects of the synthetic U12 derivatives against human hepatocellular carcinoma cell lines (HepG2) were investigated by MTT assays. As a result, U12-I, U12a-d and U12h exhibited more potent cytotoxic effects than U12 (the reference compound) [24]. Among them, U12a showed the most potent cytotoxicity against HepG2 cells (Figure 2A). Then, the cytotoxic activities of different concentrations of U12a against HepG2 cells were evaluated and compared with U12. The results showed that U12a treatment resulted in a dose-dependent inhibition on the viability of HepG2 cells over the concentration range of 0–25 µM with the IC_50_ fitted as 16.49 µM (Figure 2B), which is lower than U12 with the IC_50_ above 25 µM. Moreover, the effects of U12a and U12 on the viability of another two HCC cells lines (Huh7, SMMC-7721) were investigated. As a consequence, the cytotoxic effects of U12a were more potent than U12 in these two cell lines. Besides, the IC_50_ of the cytotoxicity of U12 against Huh7 (19.39 µM) and SMMC-7721 (>25 µM) cells was higher than HepG2 cells. 

The preliminary structure–activity relationships were discussed. For the halogen- substituted derivatives (A series), the U12-I with iodine group substituted at the C-23 showed better anti-hepatoma activity than U12-Br and U12-Cl with the Br- and Cl- groups. For the B series, the U12a-U12c with a piperazine group substituted at C-24 exhibited more potent anti-hepatoma activity than the U12d-U12i with -C(O)NH- linked groups. The derivative U12a substituted with a benzyl group exhibited more potent cytotoxic effects than U12c. Besides, U12a showed more potent effects than U12b, indicating that the cytotoxicity decreased with the growth of the carbon chain between the piperazine and benzene rings. Therefore, the piperazine ring at the 24-position is an important active group, and the length of the carbon chain between the ring and the piperazine ring is also an important factor for anti-hepatoma activity. U12d, the with -C(O)NH- linked cyclopropane groups, was more effective than U12e with the -C(O)NH- linked methylpiperazine group, which were both better than U12. The cytotoxic effects of the U12f-U12i possessing -C(O)NH- linked benzene ring, with the substituted groups of -CH_3_, -Cl, -OH and -CF_3_, ranked as U12h (-OH) > Uf (-CH_3_) > Ui, g (-Cl, -CF_3_). Thus, the -C(O)NH- linked cyclopropane and phenol groups enhanced the cytotoxic activity of the U12 derivatives.

#### 2.2.2. U12a Inhibits HepG2 Cell Proliferation by Arresting G0/G1 Phase 

**Figure 3:**
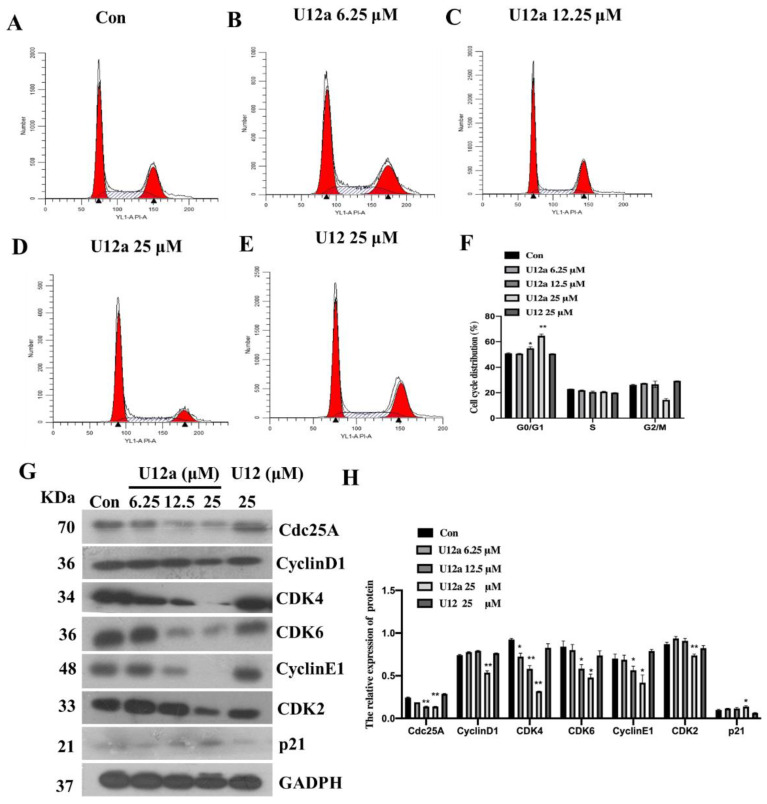
U12a inhibits HepG2 cell proliferation by arresting G0/G1 phase. (**A**–**F**) HepG2 cells were incubated with U12a (6.25,12.5,25 μM) and U12 (25 μM) for 24 h. Then all cells were harvested for further studies. Cell cycle analysis of HepG2 by flow cytometry. (**G**,**H**). Western blot analysis of Cdc25A, CyclinD1, CDK6, CDK4, CyclinE1, p21 and p27. All data represent the mean ± SD of three independent experiments. * *p* < 0.05, ** *p* < 0.01 vs. control group in the absence of any treatments.

Uncontrolled cellular proliferation is a hallmark of cancers. Blocking the cell cycle is an effective way to inhibit the proliferation of cancer cells. The cell cycle can be divided into G0/G1, S, G2, and M phases among the cell proliferation [26]. In order to verify whether U12a could induce the cell cycle arrest in HepG2 cells, flow cytometry analysis was used to determine the cell cycle distribution. As shown in Figure 3A–F, treatment of HepG2 cells with U12a resulted in a G0/G1 phase arrest, and cell population in the G0/G1 phase were increased in a dose-dependent manner, while U12 (25 μM) didn’t arrest the cell cycle (Figure 3A–F). Cyclin-dependent kinases (CDKs) and their functional cyclin partners formed a family of cyclin-CDK complexes, which play important roles in the regulation of cell cycles [27]. Studies have shown that the overexpression of CDKs and cyclins induces apoptosis of cancer cells. In cell cycle regulation, CDK4 and CDK6 form a complex with cyclin D to drive the G1 phase process. In addition, the CDK2/cyclin E complex is another important G1/S transition regulator. The cell division cycle 25A (Cdc25A) is overexpressed in various cancers and was reported to promote cell cycle progression through activating of CDKs. Studies have shown that the catalytic activities of cyclin-CDK complexes were inhibited via binding to the cyclin kinase inhibitors (cki), including p21 and p27. Among them, p21 is a universal inhibitor of CDKs, which shows critical roles in cell cycle progression through the G0-G1 phase [28,29]. Due to the above results of U12a inducing cell cycle arrest in the G0/G1 phase, Western blot analysis was further performed to determine the express levels of major cell cycle-related proteins in the G0/G1 phase. As shown in Figure 3G and H, the protein levels of Cdc25A, cyclin D1, CDK4, CDK6, cyclin E1, and CDK2 were dose-dependently decreased by the treatment of U12a. In addition, U12a upregulated the expression levels of p21 involved in G0-G1 progression. As a comparison, the levels of cell cycle-related proteins in the G0/G1 phase were not influenced by the treatment of U12 (25 μM). Thus, U12a inhibits HepG2 cell proliferation by arresting the G0/G1 phase, while U12 didn’t show any effect on cell cycle arrest at the concentration of 25 μM.

#### 2.2.3. The Pro-Apoptotic Effect of U12a on HepG2 Cells

**Figure 4:**
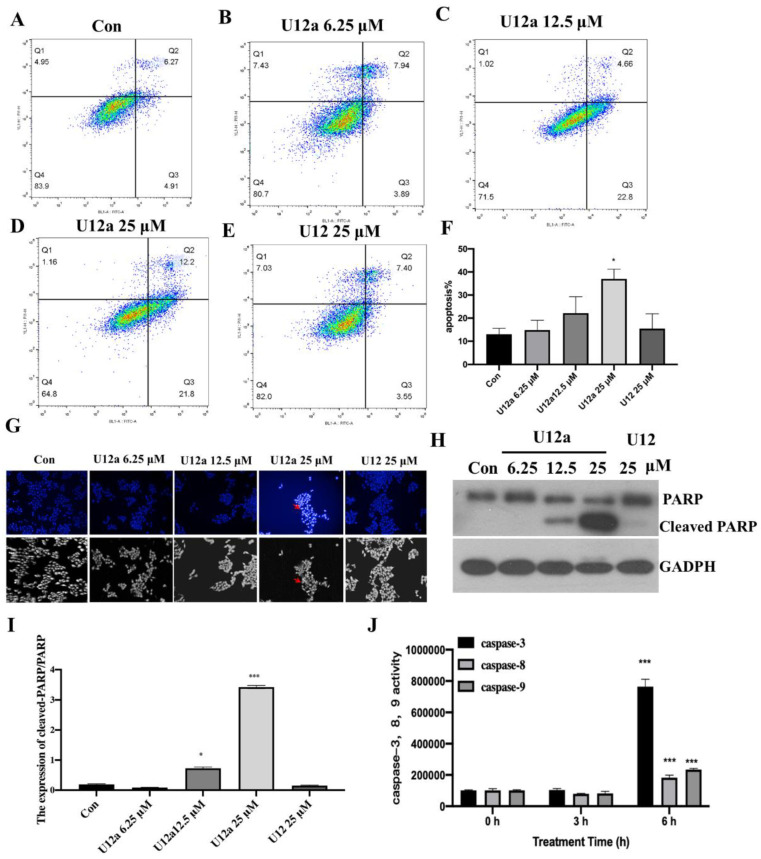
The pro-apoptosis effects of U12a on HepG2. (**A**–**F**) Flow cytometry assay to measure apoptosis of HepG2 by Annexin V-FITC/PI double staining method. HepG2 cells were treated with U12a at 0, 6.25, 12.5, and 25 μM, and U12 at 25 μM for 12 h. The cells were then stained with annexin V FITC (ANNEX300F) and ReadiDrop™ propidium iodide (1351101), and analyzed on a flow cytometer. The rate of early and advanced apoptosis can be seen in the bottom right quadrant (E3) and top right quadrant (E2), respectively. Dead cells can be seen in the top left quadrant (E1). Healthy cells are in the bottom left quadrant (E4). (**G**) Digital phase contrast and fluorescent micrographs of cells before and after U12a (6.25, 12.5, 25 μM) or U12 (25 μM) treatment for 12 h. Cell nuclei were stained with Hochest 33528. Apoptotic cells are labeled by red arrows. (**H**,**I**) PARP and cleaved PARP expression levels in HepG2 cells upon treatment of U12a (6.25, 12.5, 25 μM) or U12 (25 μM). (**J**)The activities of caspase-3, -8, and -9, analyzed by caspase kits after treatment of U12a (25 µM) for 6h. * *p* < 0.05 vs. control group. *** *p* < 0.001 compared with control or 0 h group.

Apoptosis, a highly regulated process of programmed cell death, plays an important role in eliminating damaged or aberrant cells [30]. The flow cytometry assay was used to explore whether U12a has pro-apoptotic effects. As show by the Annexin V-FITC/PI double staining results, the percentage of apoptotic cells significantly increased from 13.0% (control) to 14.8% (6.25 μM), 22.2% (12.5 μM) and 37.0% (25 μM) after treatment of U12a for 12h (Figure 4A–F). Furthermore, the induction of apoptosis by U12a was confirmed by hoechst 33258 staining (Figure 4G). Poly (ADP-ribose) polymerase (PARP) cleavage is considered as one of the biomarkers for the detection of apoptosis. To determine the underlying apoptotic mechanism, the expression of cleaved-PARP was detected by Western blot analysis. As shown in Figure 4H –I, U12a induced PARP cleavage in HepG2 cells in a dose-dependent manner. As a comparison, U12 didn’t show apoptotic effects at the concentration of 25 μM (Figure 4A–H). The caspase signaling pathway plays a crucial role in cell apoptosis. In order to further study whether U12a induces the apoptosis of HepG2 cells by activating the caspase signaling pathway, the caspase activation activities, including caspase-3, -8, and -9, were detected by caspase kits. Caspase-3 is the apoptosis executioner, which is activated by initiator caspase proteins, caspase-8 and caspase-9. As shown in Figure 4J, the activities of caspase-3, -8, and -9 were all increased after the treatment of U12a (25 µM) for 6h. Among them, the activity of caspase-3 was significantly enhanced. Thus, U12a might activate the caspase signaling pathway to induce apoptosis.

#### 2.2.4. U12a Suppressed the Activation of the PI3K/AKT/mTOR Pathway

**Figure 5:**
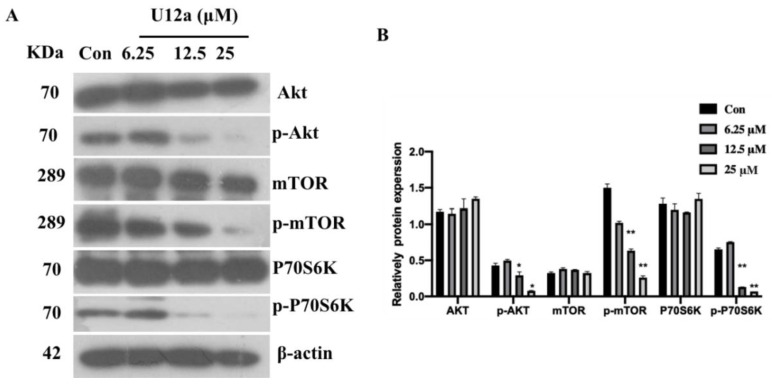
Effect of U12a on the PI3K/AKT/mTOR pathway in HepG2 cell. (**A**) Protein expression levels of AKT, p-AKT, mTOR, p-mTOR, p70S6k, p-p70S6k and β-actin in HepG2 cells with Ua12 (6.26, 12.5, 25 μM) for 24 h. (**B**) The bar graph describes the relative gray values of related proteins that were calculated using image J. All data represent the mean ± SD of three independent experiments. * *p* < 0.05, ** *p* < 0.01 compared with the Con group.

The PI3K/AKT/mTOR pathway plays a pivotal role in the pathogenesis of hepatocellular carcinoma (HCC). The activation of the PI3K/AKT/mTOR pathway has been reported in 40–50% of HCC [31]. Therefore, the effects of U12a on the PI3K/AKT/mTOR pathway in HepG2 cells were investigated. After treating with increasing concentrations of U12a for 24 h, the phosphorylation levels of AKT and its downstream factor mTOR in HepG2 cells were effectively suppressed in a dose-dependent manner (Figure 5A,B). In addition, mTOR positively regulated the phosphorylation of ribosomal p70S6 kinase (p70S6k), which subsequently lead to mTOR-dependent gene transcription that regulated cell proliferation. Thus, we investigated the effect of U12a on the phosphorylation of p70S6k. As a result, U12a dose-dependently decreased the phosphorylation of p70S6k (Figure 5A,B). Consequently, Ua12 suppressed the activation of the PI3K/AKT/mTOR pathway, leading to the inhibition of HepG2 cells proliferation. 

#### 2.2.5. U12a Inhibited Tumor Growth in HepG2 Xenograft Model

**Figure 6:**
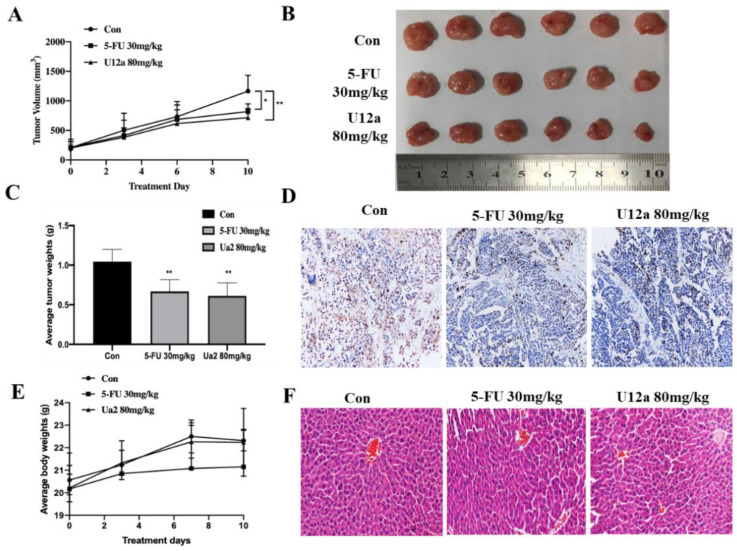
In vivo effects of U12a in the hepatocellular carcinoma (HCC) nude mouse xenograft model. (**A**) Average tumor volume of different groups (0, 5-FU 30 mg/kg, U12a 80 mg/kg). (**B**) Tumor size and weight of different groups. (**C**) Average tumor weight of different groups. (**D**) Tumors were excised and processed for immunostaining for Ki-67. (**E**) Average body weight of nude mice. (**F**) H&E staining of liver from different groups (0, 5-FU 30 mg/kg, U12a 80 mg/kg). Data represented mean ± SD (*n* = 6). * *p* < 0.05, ** *p* < 0.01 compared to control.

The above studies indicated that U12a inhibited the proliferation of HepG2 cells in vitro. The in vivo anti-cancer efficacy of U12a was further investigated by the nude mouse xenograft model. U12a was administered intraperitoneally at the dose of 80 mg/kg every day. 5-FU (30 mg/kg) was used as a positive control. After 10 days of treatment, mice were sacrificed and the tumors were collected. As shown in Figure 6A–C, U12a administration suppressed the tumor growth and resulted in shrinkage of the tumor size at the dose of 80 mg/kg for 10 days, compared with the control group. The inhibition effects of U12a (80 mg/kg) on tumor growth was almost equivalent to the positive control 5-FU (30 mg/kg). Furthermore, U12a decreased the expression of Ki-67 in tumor issues, a cell proliferation marker, compared with the control group (Figure 6D). Besides, there was no difference in body weight of nude mice in the U12a treatment group compared with the control group, and both had an increasing trend, while the body weight of 5-Fu treatment group possessed obvious side effects with a decreasing trend (Figure 6E). The livers were then processed and subjected to H&E staining. As shown by representative images in Figure 6F, U12a treatment did not cause any obvious abnormality or organ damage compared with the control group. The above results indicated that U12a inhibits the growth of xenograft liver tumors in vivo without observable adverse effects.

## 3. Materials and Methods

### 3.1. Chemistry

#### General

NMR spectra were acquired on Bruker Avance 600III spectrometers (Bruker, Germany) with tetramethylsilane as the internal standard, using CDCl_3_ as solvent. HRESIMS experiments were conducted on a Thermo Scientific Q Exactive Quadrupole-Orbitrap mass spectrometer (Thermo, Waltham, MA, USA). Column chromatography was performed on silica gel (200−300 mesh, Qingdao Marine Chemical Inc., Qingdao, China). Analytical HPLC was performed on a Shimadzu LC-20AT pump system (Shimadzu, Japan) with a DAD, semipreparative was performed on a Shimadzu LC-8A pump system (Shimadzu, Japan) with a DAD. Fractions were monitored by TLC (Qingdao Marine Chemical Inc., Qingdao, China) and visualized under a UV lamp at 254 nm or 365 nm after spraying with 5% H_2_SO_4_ in ethanol, followed by heating. Reagents and materials were obtained from commercial suppliers and were used without further purification.

### 3.2. Synthesis

#### 3.2.1. General Procedure for 3α, 7 β-Diacetoxyursodeoxycholic Acid (U-I)

A solution of UDCA (1.0 g, 2.55 mmol) and 4-DMAP (100 mg, 0.8 mmol) in EtOAc (100 mL) and Py (10 mL) was stirred until dissolved completely, then Ac_2_O (12 mL) was added dropwise. After the addition was complete, the mixture was stirred and reacted at room temperature for 10 h. The organic layer was extracted with H_2_O (100 mL) and EtOAc (100 mL). The combined organic layer was washed with 1 mol/L HCl (100 mL) and saturated NaCl (100 mL) solution, dried (Na_2_SO_4_), and concentrated under vacuum to yield U-I as a colorless oil (98% yield).

#### 3.2.2. General Procedure for U12-Cl, U12-Br and U12-I

U-I (200 mg, 0.42 mmol) was added to SOCl_2_ (5 mL), and the mixture was stirred at 40 °C for 1 h. Then, the mixture was heated to 70 °C and Br/Py/SOCl_2_ (55 µL/70 µL/400 µL) mixture was added dropwise. After dripping, the reaction mixture was stirred for 6 h. The solvent was removed under vacuum, the residue was dissolved in CH_2_Cl_2_ and was added to MeOH in ice-bath, concentrated under vacuum. The crude product was purified by preparative HPLC to yield U12-Cl and U12-Br. A solution of U12-Br (20 mg) in acetone (2 mL) was stirred until dissolved completely. Then, NaI and Bu_4_N^+^I^−^ dissolved in acetone (2 mL) was added dropwise. The reaction mixture was stirred at room temperature for 6 h. The organic layer was extracted with H_2_O (3 mL) and EtOAc (10 mL), dried (Na_2_SO_4_), and concentrated under vacuum to yield U12-I. 

#### 3.2.3. General Procedure for U12a-U12i

U-I (200 mg, 0.42 mmol) was added to a stirred solution of DMF (10 mL). After completely dissolving, HOBt (30 mg, 2.21 mmol) and EDC·HCl (46 mg, 2.21 mmol) dissolved in DMF were added dropwise under nitrogen atmosphere in ice-bath. The reaction mixture was stirred for 1 h to form an intermediate. Then, benzylpiperazine, trans-1-cinnamylpiperazine, piperazine, cyclopropylamine, 1-amino-4-methyl piperazine, 4-Aminotoluene, p-Chloroaniline, 4-aminophenol or 4-Aminobenzotrif luoride (0.5 mmol) was added, and the reaction was continued for 24 h. Then, the organic layer was extracted with H_2_O (50 mL) and EtOAc (50 mL), and concentrated under vacuum. The crude product was purified by chromatography on silica gel to yield U13a-U13i. U13a-i (0.11 mmol) were respectively dissolved in THF (8 mL), and then NaOH (16 mg, 0.4 mmol) that was dissolved in MeOH (1 mL) was added. The mixture was stirred for 1 h at room temperature. Then, the organic layer was extracted with 1 mol/L HCl (20 mL) and EtOAc (20 mL). The combined organic layer was washed with saturated NaCl solution (20 mL), dried by Na_2_SO_4_, and concentrated under vacuum. The crude product was purified by chromatography on silica gel to yield U12a-U12i. 

#### 3.2.4. Analytical Data

The ^1^H NMR, ^13^C NMR and ESI-MS data of all final compounds, U-I, U12-Cl, U12-Br, U12-I and U12a-U12i, are present in Supplementary Material. The spectra ^1^H NMR, ^13^C NMR and mass are also enclosed.

### 3.3. Cell Viability Assay

The cell viability was monitored using MTT assay. HepG2 cells were seeded in a 96-well plate at a density of 1 × 10^4^ cells/well. The cells were grown overnight at 37 °C in humidified 5% CO_2_ and then treated with compounds, including the synthetic U12 derivatives and U12 in a final concertation of 25 μM for 24 h. Supernatants were discarded and the cells were incubated, with 75μL of fresh medium added with 15μL methylthiazolyldiphenyl-tetrazolium bromide (MTT) solution (GIBICOL, USA), for 4 h at 37 °C. Then, the media containing MTT were removed and the dark-blue formazan crystals were dissolved by 150 mL of DMSO. The absorbance was recorded using a microplate reader at an optical density (OD) of 490 nm. The measured OD values were converted into cell viability as follows: cell viability ratio (%) = (A_treatment_ − A_blank_)/(A_control_ − A_blank_) × 100%. 

### 3.4. Flow Cytometry Assay for Cell Cycle Detection 

HepG2 cells were plated on 6-well plates and treated with different concentrations of U12a (0, 6.25, 12.5, 25 μM) for 24 h. Cells were trypsinized with 0.25% trypsin without EDTA, and washed with PBS. Then the cells were harvested and fixed with 70% ethanol at 4 °C overnight. After washing twice with PBS, the cells were stained with a solution of 100 μL RNase A and 400 μL Propidium Iodide (PI) for 30 min in the dark at room temperature. At least 10,000 cells were recorded for each sample. The percentage of cells in G1/G0, S and G2/M phases was detected by a BD FACS Calibur flow cytometer. 

### 3.5. Annexin V-FITC/PI Double Staining Assay for Cellular Apoptosis Detection

HepG2 cells were seeded at a density of 2 × 10^5^ cells/well in 6-well plates. After treatment with U12a (0, 6.25, 12.5, 25 μM) for 12 h, cells were collected into tubes and suspended in 1 × binding buffer at a concentration of 1 × 10^5^ cells/mL. The medium (100 mL), with 1 × 10^5^ cells, was mixed with 5 mL of Annexin V-FITC and 5 mL of propidium iodide (BD Biosciences, San Jose, CA, USA). The mixed solution was gently vortexed and incubated for 15 min in the dark at room temperature. The cell apoptosis analysis was carried out by flow cytometry (BD FACS Calibur) within 1 h. 

### 3.6. Hochest 33528 Staining

The HepG2 cells were plated in 6-well plates at a density of 1 × 10^5^ cells/mL, and grew overnight. Then, different concentrations of U12a (0, 6.25,12.5 and 25 μM) were treated for 24 h. The cells were harvested by trypsinization, washed twice with PBS, fixed in 4% formaldehyde for 30 min, and then stained with 1 µM Hoechst 33258 (Fisher Scientific, Waltham, MA, USA). Fluorescence microscopy (High-Content Screening; Perkin-Elmer, Waltham, MA, USA) with a filter for Hoechst (350/461 nm) was used to detect the change in nuclear morphology.

### 3.7. Western Blot Assay

Western blotting was performed to detect the expression of the related proteins in HepG2 cells. The cells were harvested and total proteins were isolated in 1% sodium dodecyl sulphate (SDS). The lysed solution was centrifuged at 12,000 rpm for 15 min at 4 °C. Subsequently, the protein concentrations were assessed using the BCA protein assay. Samples with the same amount of total protein were subjected to around 8%–10% sodium dodecylsul phatepolyacrylamide gel electrophoresis (SDS-PAGE) and transferred onto PVDF membranes (Millipore, Boston, MA, USA). The membranes were blocked with TBST containing 5% non-fat milk or 5% Bovine serum albumin (BSA) for at least 1 h at room temperature, and incubated with the primary antibodies mTOR(1:1000), p-mTOR(1:1000), p70S6k(1:1000), p-p70S6k (1:1000), CyclinD1(1:1000), Cdc25A(1:1000), p27(1:1000), CDK4(1:1000), CDK6(1:1000), PARP and β-actin (1:1000) at 4 °C overnight. Then, they were incubated with the corresponding secondary antibodies (dilution, 1:4000) for a minimum of 1 h at room temperature. After washing with TBST three times, the expression of each protein in the membrane was detected by enhanced chemiluminescence reagent (Advansta, USA). The bands were scanned and the data were quantified by densitometric analysis using Image J software (National Institutes of Health, Bethesda, MD, USA).

### 3.8. In vivo Anti-tumor Activity Detected by HepG2 Xenograft Model

The animal experiments were conducted under an approved protocol of the Institutional Animal Care and Use Committee of Xiamen University. Female nude mice (BALB/c nu/nu, 6 weeks old) were provided by Beijing Vital River Laboratory Animal Technology Co., Ltd. HepG2 cells (2.5 × 10^6^ cells/0.1 mL) were injected subcutaneously into the left flank of nude mice to establish a liver tumor xenograft animal model. When tumor volume reached ∼200 mm^3^ (about 8 days), the nude mice were randomly divided into three groups: negative control group (0.2% DMSO), positive control group (5-FU 30 mg/kg), and experimental group (U12a 80 mg/kg). Drugs were administered for 10 days, then the mice were sacrificed and their tumor tissues, as well as liver, were harvested. The tumor was fixed with formalin, embedded, and immunostained for Ki-67. The livers from different groups were tested with H&E staining. Tumors’ volumes were calculated as following: volume(mm^3^) = 0.5 × length × width^2^. 

### 3.9. Statistical Analyses

All data represent the mean ± SD of three independent experiments, and was analyzed by GraphPad Prism Software version 9.0 (GraphPad Software Inc., La Jolla, CA, USA). Statistical significance was calculated using one-way analysis of variance (ANOVA) followed by Dunnett’s test and * *p* < 0.05 was considered statistically significant.

## 4. Conclusions

UDCA is a widely used therapeutic agent in cholestatic liver disease. In our previous study, U12, a derivative of UDCA, showed effective anti-hepatoma activities. However, the low polarity and large doses limited the druglikeness of U12. In order to find the derivatives endowed with more potent pharmacological profiles, the structural modification and optimization of U12 were further investigated. In this study, twelve U12 derivatives of two series (A and B) were synthesized by substitution, esterification and amidation reactions. The A series were halogen-substituted derivatives, and the B series were conjugates of UDCA with nitrogen heterocycles, cyclopropane or substituted benzene rings by N-acetyl moiety. The synthesis, cytotoxic activities, pro-apoptotic mechanism and structure–activity relationship were further investigated. The evaluation of the cytotoxic activities of synthetic derivatives against HepG2 indicated that the U12 derivatives, except for U12g and U12i, showed more effective cytotoxicity on HepG2 cells than U12. Among them, U12a exhibited the most potent anti-hepatocellular carcinoma activity. The preliminary structure–activity relationships imply that the piperazine ring at the 24-position is an important active group, as well as the length of the carbon chain between the ring and the piperazine ring also being an important factor for anti-hepatoma activity. Mechanism studies indicated that U12a inhibited HepG2 cell proliferation by arresting the G0/G1 phase, and suppressed the activation of the PI3K/AKT/mTOR pathway. Besides, U12a induced HepG2 cells apoptosis through activating the caspase signaling pathway. Furthermore, U12a evidently inhibits the growth of HepG2-derived tumor xenografts in vivo without observable adverse effects. Thus, U12a might be considered as a promising candidate for the treatment of hepatocellular carcinoma.

## Data Availability

Data is contained within article and Supplementary Materials.

## Supplementary Materials

The following are available online at https://www.mdpi.com/article/10.3390/ph15010107/s1: the ^1^H NMR, ^13^C NMR and ESI-MS data, and spectra of all final compounds U-I, U12-Cl, U12-Br, U12-I and U12a-U12i.
