# Supplementary material for: Synthesis and Anti-Hepatoma Activities of U12 Derivatives Arresting G0/G1 Phase and Inducing Apoptosis by PI3K/AKT/mTOR Pathway
Source: Pharmaceuticals (Basel). 2022 Jan 17;15(1):107. doi: 10.3390/ph15010107 (PMC8781819; doi:10.3390/ph15010107)

Supplementary Material

Synthesis and anti-hepatoma activities of U12 derivatives arresting G0/G1 phase and inducing apoptosis by PI3K/Akt/mTOR pathway

Renjing Yang, [†,‖] Chunchun Du, [†,‖] Ting Cao, [†,‖] Guanghui Wang,[†] Xin Jiang,[†] Jun Gao,[†] Ting Lin, [†]Cuiling Sun,[†] Rong Ding,[†] Wenjing Tian,[*, †] Haifeng Chen[*,†]

[†]Fujian Provincial Key Laboratory of Innovative Drug Target, School of Pharmaceutical Sciences, Xiamen University, Xiamen 361005, P. R. China.

[*]Author to whom correspondence should be addressed; E-Mail: tianwj@xmu.edu.cn, haifeng@xmu.edu.cn

[‖]These authors contributed equally to this research.

Index:

1. ^{1}H and ^{13}C NMR data of intermediates U13a-U13i
2. ^{1}H and ^{13}C NMR data of U12-Cl, U12-Br, U12-I
3. ^{1}H and ^{13}C NMR data of U12a-U12i
4. ^{1}H ,^{13}C NMR and ESI-MS data of U12 derivatives

1. ^{1}H and ^{13}C NMR data of intermediates U13a-U13i

1.1. (3R,7S,10S,13R)-17-((R)-5-(4-benzylpiperazin-1-yl)-5-oxopentan-2-yl)-10,13-dimethyl-hexadecahydro-1H-cyclopenta[a]phenanthrene-3,7-diyl diacetate (U13a).Yellow oil (31% yield).^{1}H-NMR (600MHz, $CDCl_3$) δ_H: 7.33 (3H, d, *J*= 3.6Hz), 7.28 (2H, d, *J*= 3.6 Hz), 4.76 (1H, m), 4.66 (1H, m), 2.02 (3H, s), 1.9(3H, s), 0.97 (3H, s), 0.92 (3H, d), 0.67 (3H, s); ^{13}C-NMR (150MHz, $CDCl_3$)δ_C: 172.1 170.8, 170.7, 133.7, 129.4 (2×C), 128.6 (2×C), 127.5, 73.7, 73.7, 63.0, 60.5, 55.4, 55.2, 53.2, 52.9, 43.7, 42.2, 40.1, 40.1, 39.5, 35.6, 34.6, 34.1,33.0 (2×C), 31.5, 28.6, 26.5, 25.8, 23.4, 22.0, 21.5, 21.3, 18.7, 12.2; HR-ESI-MS: *m/z* 635.4418 $[M+H]^+$.

1.2. (3R,7S,10S,13R)-17-((R)-5- (4-cinnamylpiperazin-1-yl)-5-oxopentan-2-yl)-10,13-dimethylhexadecahydro-1H-cyclopenta[a]phenanthrene-3,7-diyldiacetate(U13b). Yellow oil (33% yield). ^{1}H-NMR (600MHz, $CDCl_3$) δ_H: 7.38 (2H, d, *J*= 7.8 Hz), 7.24 (2H, d, *J*= 7.2 Hz), 4.76 (1H, m), 4.66 (1H, m), 1.98 (3H, s), 0.97 (3H, s), 0.94 (3H, d), 0.67 (3H, s); ^{13}C-NMR (150MHz, $CDCl_3$) δ_C: 172.2, 170.8, 170.7, 136.7, 128.8 (4×C), 127.9, 126.5 (2×C), 73.7, 73.7, 61.0, 55.4, 55.2, 52.9, 43.7, 42.2, 40.1, 40.1, 39.5, 35.6, 34.6, 34.1, 33.0 (2×C), 31.5, 30.3, 28.6, 26.5, 25.8, 23.4, 22.0, 21.5, 18.8, 12.2; HR-ESI-MS: *m/z* 661.4572 $[M+H]^+$.

1.3. (3R,7S,10S,13R)-10,13-dimethyl-17-((R)-5-oxo-5-(piperazin-1-yl) pentan-2- yl) hexadecahydro-1H-cyclopenta[a]phenanthrene-3,7-diyldiacetate(U13c). White power (21% yield). ^{1}H-NMR (600MHz, $CDCl_3$) δ_H: 4.76 (1H, m), 4.66 (1H, m), 2.02 (3H, s), 0.97 (3H, s), 0.94 (3H, d), 0.68 (3H, s); ^{13}C-NMR (150MHz, $CDCl_3$) δ_C: 172.2, 170.8, 170.7, 73.7, 73.7, 55.4, 55.1, 44.2, 44.0, 43.8, 42.2, 40.1, 40.1, 39.5, 35.5, 34.6, 34.1, 33.0 (2×C), 31.3, 30.1, 28.7, 26.5, 25.8, 23.4, 22.0, 21.5, 21.3, 18.7,12.2; HR-ESI-MS: *m/z* 545.3950 $[M+H]^+$.

1.4 (3R,7S,10S,13R)-17-((R)-5-(cyclopropylamino)-5-oxopentan-2-yl)-10,13-dimethylhexadecahydro-1H-cyclopenta[a]phenanthrene-3,7-diyldiacetate(U13d). White power (37% yield). ^{1}H-NMR (600MHz, $CDCl_3$) δ_H: 4.76 (1H, m), 4.66 (1H, m), 2.02 (3H, s), 1.98 (3H, s), 0.97 (3H, s), 0.91 (3H, d), 0.67 (3H, s); ^{13}C-NMR (150MHz, $CDCl_3$) δ_C: 175.0, 170.8, 170.7, 73.7, 73.7, 55.4, 55.2, 43.7, 42.2, 40.1, 40.1, 39.5, 35.5, 34.6, 34.1, 33.6, 33.0 (2×C), 31.8, 28.6, 26.5, 25.7, 23.4, 22.0, 21.5, 21.3, 18.6,12.2, 6.8, 6.8; HR-ESI-MS: *m/z* 538.3506 $[M+Na]^+$.

1.5. (3R,7S,10S,13R)-10,13- dimethyl-17-((R)-5-((4-methylpiperazin-1-yl)amino)-5-oxopentan-2-yl)hexadecahydro-1H-cyclopenta[a]phenanthrene-3,7-diyldiacetate (U13e). White power (18% yield). ^{1}H-NMR (600MHz, $CDCl_3$) δ_H: 4.76 (1H, m), 4.66 (1H, m), 2.02 (3H, s), 1.98 (3H, s), 0.97 (3H, s), 0.92 (3H, d), 0.68 (3H, s); ^{13}C-NMR (150 MHz, $CDCl_3$) δ_C: 171.4, 170.8, 170.7, 73.7, 73.7, 55.9 (2×C), 55.3, 55.2, 53.6 (2×C), 43.7, 42.2, 40.1, 40.1, 39.5, 35.6, 34.6, 34.6, 34.1, 33.0 (2×C), 32.1, 31.8, 28.6, 26.5, 25.7, 23.4, 22.0, 21.5, 21.3, 18.8, 12.2; HR-ESI-MS: *m/z* 574.4224 $[M+H]^+$.

1.6. (3R,7S,10S,13R)-10,13-dimethyl-17-((R)-5-oxo-5-(p-tolylamino) pentan-2-yl) hexadecahydro-1H-cyclopenta[a]phenanthrene-3,7-diyl diacetate (U13f). White power (46% yield). ^{1}H-NMR(600MHz, $CDCl_3$) δ_H: 7.39(2H, d, *J*= 7.8 Hz), 7.11 (2H, d, *J*= 8.4 Hz), 4.76 (1H, m), 4.66 (1H, m), 2.30 (3H, s), 2.02 (3H, s), 1.98 (3H, s), 0.97 (3H, s), 0.95 (3H, d), 0.68 (3H, s); ^{13}C-NMR(150MHz, $CDCl_3$) δ_C: 171.7, 170.8, 170.7, 135.6, 133.9, 129.6(2×C), 120.0(2×C), 73.7, 73.7, 55.3, 55.2, 43.7, 42.2, 40.1, 40.1, 39.5, 35.4, 34.7, 34.6, 34.1, 33.0(2×C), 31.7, 28.6, 26.5, 25.8, 23.4, 22.0, 21.5, 21.3, 21.0, 18.7,12.2; HR-ESI-MS: *m/z* 588.3666 $[M+Na]^+$.

1.7 (3R,7S,10S,13R)-17-((R)-5-((4-chlorophenyl) amino)-5-oxopentan-2-yl)-10,13-dimethylhexadecahydro-1H-cyclopenta[a]phenanthrene-3,7-diyl diacetate (U13g). Whit

e power (43% yield). ^{1}H-NMR (600MHz, $CDCl_3$) δ_H: 7.48 (2H, d, *J*= 9.0 Hz), 7.27 (2 H, d, *J*= 8.4 Hz), 4.76 (1H, m), 4.66 (1H, m), 1.98 (3H, s), 0.97 (3H, s), 0.96 (3H, d), 0.68 (3H, s); ^{13}C-NMR (150MHz, $CDCl_3$) δ_C: 171.8, 170.8, 170.7, 136.7, 129.2, 129.1 (2×C), 121.1 (2×C), 73.7, 73.7, 55.3, 55.2,43.7, 42.2, 40.1, 40.1, 39.5, 35.4, 34.6 (2×C), 34.1, 33.0 (2×C), 31.6, 28.6, 25.7,23.4, 22.0, 21.3, 18.7, 12.2; HR-ESI-MS: *m/z* 608.3121 $[M+Na]^+$.

1.8. (3R,7S,10S,13R)-17-((R)-5-((4-hydroxyphenyl)amino)-5-oxopentan-2-yl)-10,13-dimethylhexadecahydro-1H-cyclopenta[a]phenanthrene-3,7-diyl diacetate (U13h).White power (46% yield). ^{1}H-NMR (600MHz, $CDCl_3$) δ_H: 7.29 (2H, d, *J*= 8.4Hz), 6.76 (2H, d, *J*= 8.4 Hz), 4.76 (1H, m), 4.66 (1H, m), 2.03 (3H, s), 1.98 (3H, s), 0.96 (3H, s), 0.94 (3H, d), 0.66 (3H, s); ^{13}C-NMR(150MHz, $CDCl_3$)δ_C: 172.5, 171.2, 170.9, 153.4, 130.4, 122.6 (2×C), 115.9 (2×C), 73.9, 73.8, 55.3, 54.9, 43.7, 42.1, 40.1, 40.0, 39.5, 35.4, 34.6, 34.2, 33.0, 33.0, 31.9, 28.6, 26.5, 25.7, 23.3, 22.0, 21.6, 21.3, 18.7, 12.2;HR-ESI-MS: *m/z* 590.3462 $[M+Na]^+$.

1.9 (3R,7S,10S,13R)-10,13-dimethyl-17-((R)-5-oxo-5-((4-(trifluoromethyl) phen-yl) amino) pentan-2-yl) hexadecahydro-1H-cyclopenta[a]phenanthrene-3,7-diyl diacetate (Ui1). White power (43% yield). ^{1}H-NMR (600MHz, CD_3OD) δ_H: 7.77 (2H, d, *J*= 8.4 Hz), 7.60 (2H, d, *J*= 8.4 Hz), 4.77 (1H, m), 4.64 (1H, m), 2.02 (3H, s), 1.97 (3H, s), 1.03 (3H, d), 1.01 (3H, s), 0.74 (3H, s); ^{13}C-NMR (150MHz, CD_3OD) δ_C: 175.4, 172.6, 172.5, 143.6, 127.0, 127.0, 120.7(4×C), 75.2, 75.1, 56.6,56.4, 44.8, 43.4, 41.3, 41.2, 40.7, 36.8, 35.5, 35.1, 35.0, 33.9, 33.9, 33.0, 29.5,26.9, 23.6, 22.3, 21.8, 21.2, 19.0, 12.5; HR-ESI-MS: *m/z* 642.3373 $[M+Na]^+$.

2. ^{1}H and ^{13}C NMR spectrum of U12 derivatives

methyl (4R)-4-((3R,7S,10S,13R)-7-acetoxy-3-hydroxy-10,13-dimethylhexadecahydr-o-1H-cyclopenta[a]phenanthren-17-yl)-2-chloropentanoate (^{1}H) U12-Cl:

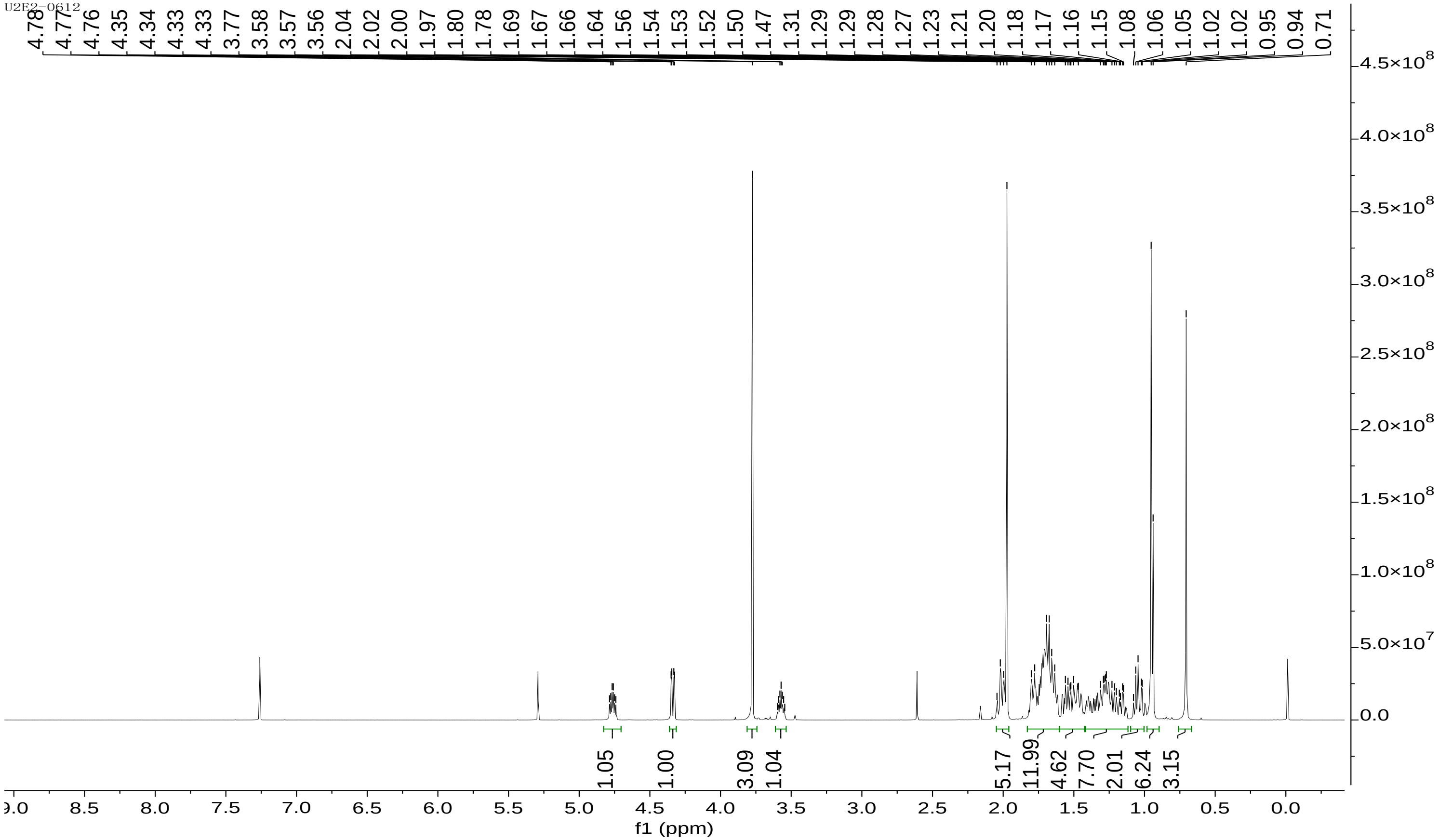

methyl (4R)-4-((3R,7S,10S,13R)-7-acetoxy-3-hydroxy-10,13-dimethylhexadecahydr-o-1H-cyclopenta[a]phenanthren-17-yl)-2-chloropentanoate (^{13}C) U12-Cl:

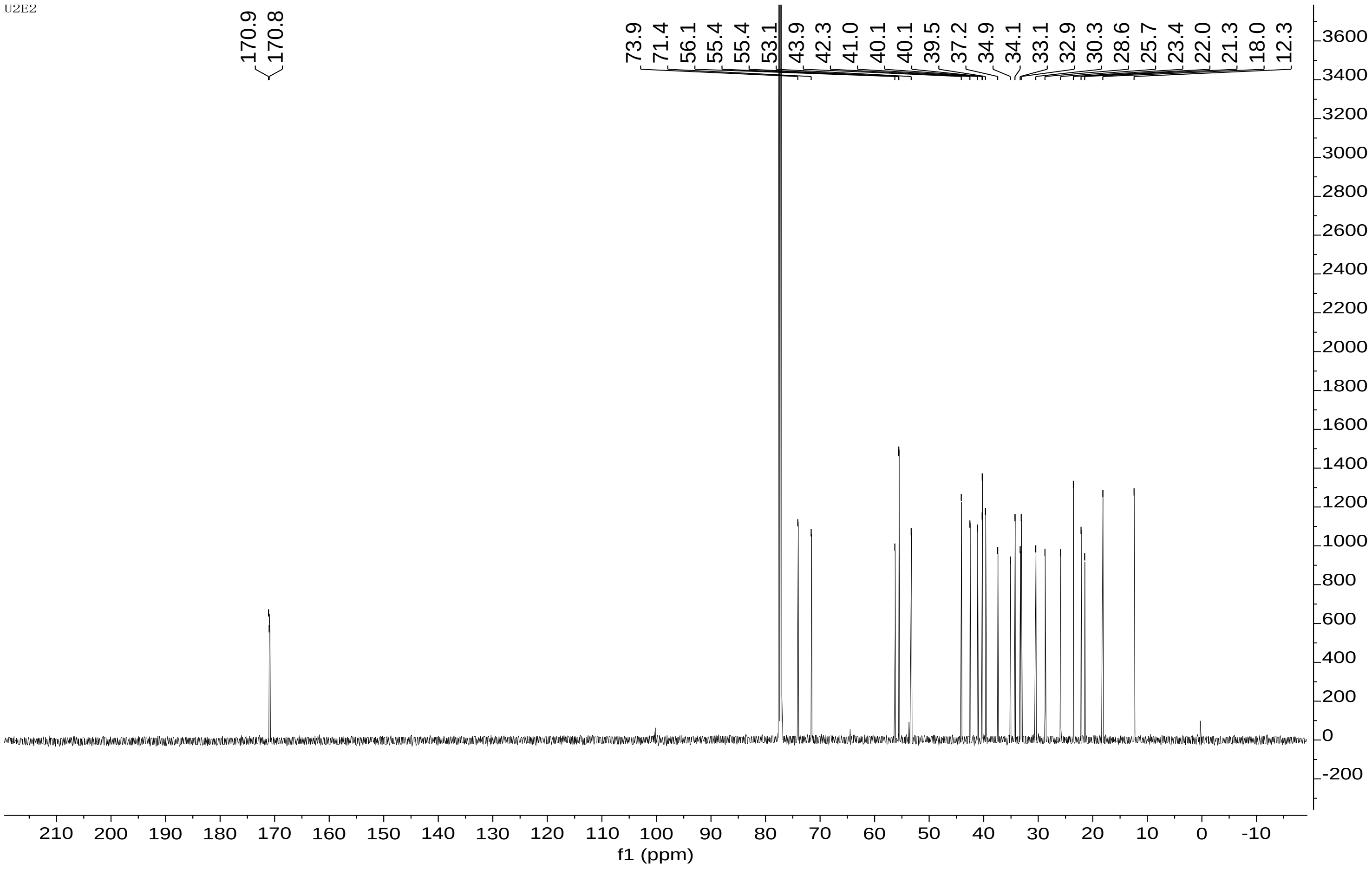

methyl (4R)-4-((3R,7S,10S,13R)-7-acetoxy-3-hydroxy-10,13-dimethylhexadecahydr-o-1H-cyclopenta[a]phenanthren-17-yl)-2-chloropentanoate (MS) U12-Cl:

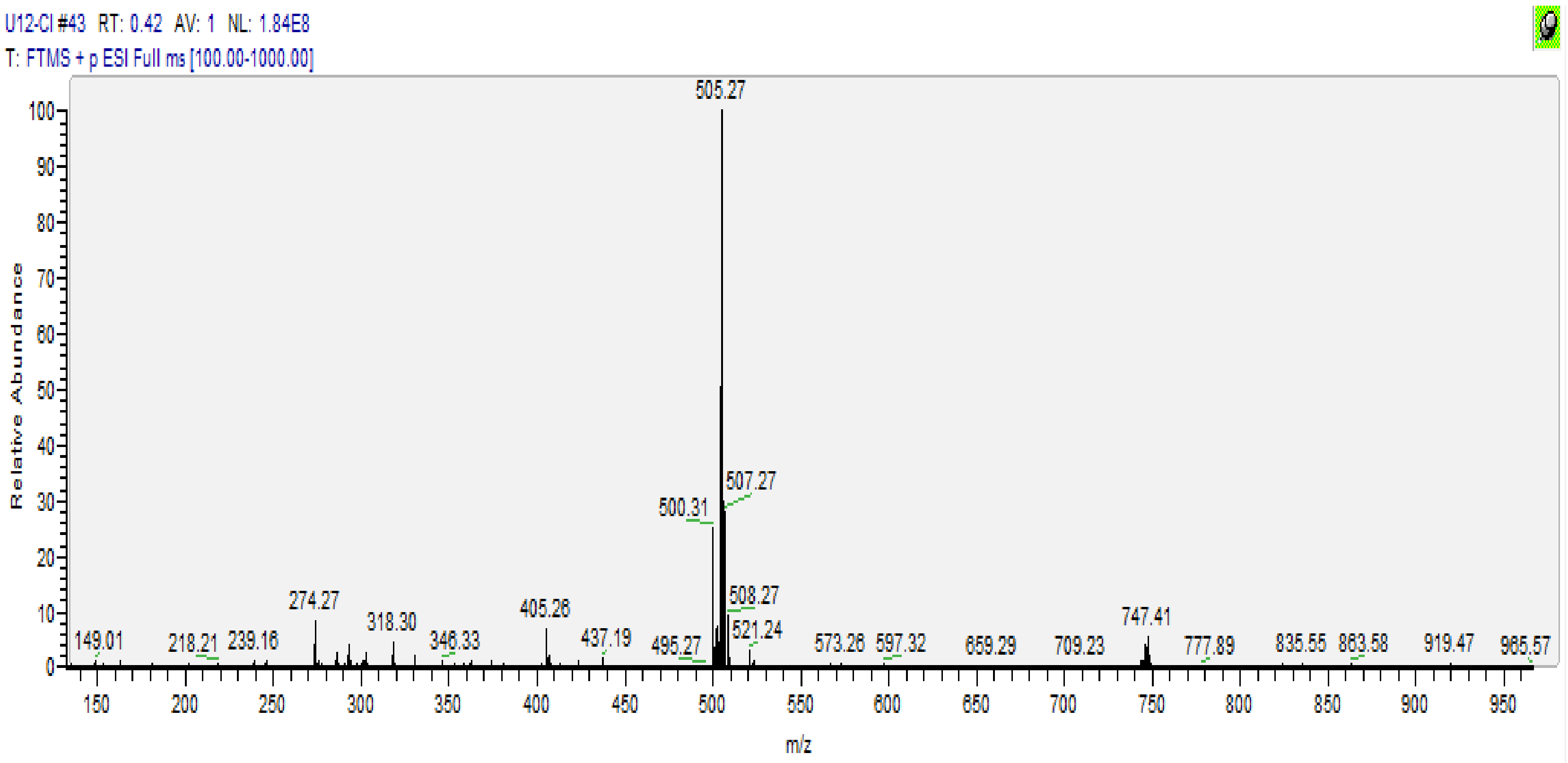

methyl (4R)-4-((3R,7S,10S,13R)-7-acetoxy-3-hydroxy-10,13-dimethylhexa-decahydr-o-1H-cyclopenta[a]phenanthren-17-yl)-2-bromopentanoat e (^{1}H) U12-Br:

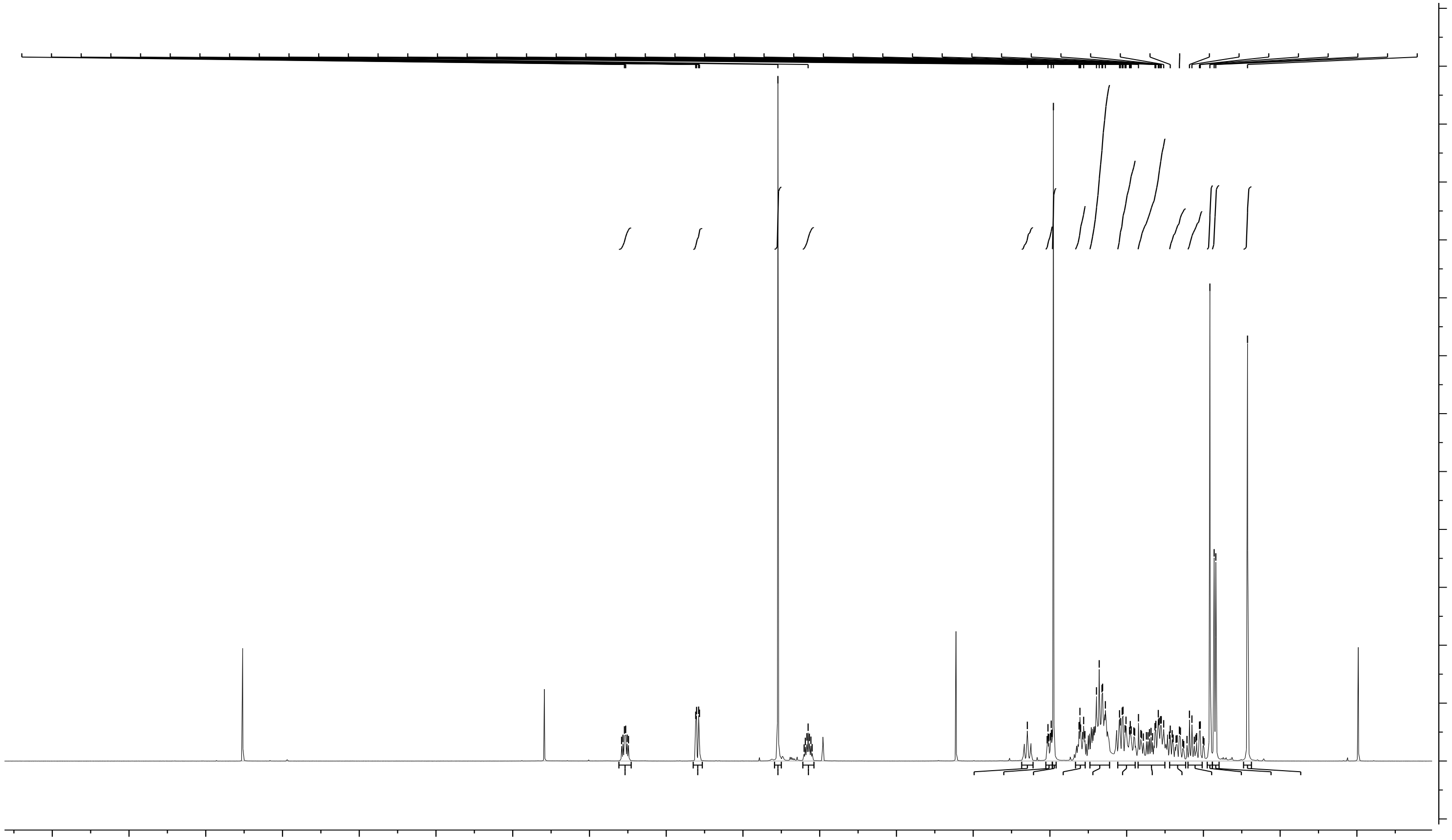

methyl (4R)-4-((3R,7S,10S,13R)-7-acetoxy-3-hydroxy-10,13-dimethylhexa-decahydr-o-1H-cyclopenta[a]phenanthren-17-yl)-2-bromopentanoat
e (^{13}C) U12-Br:

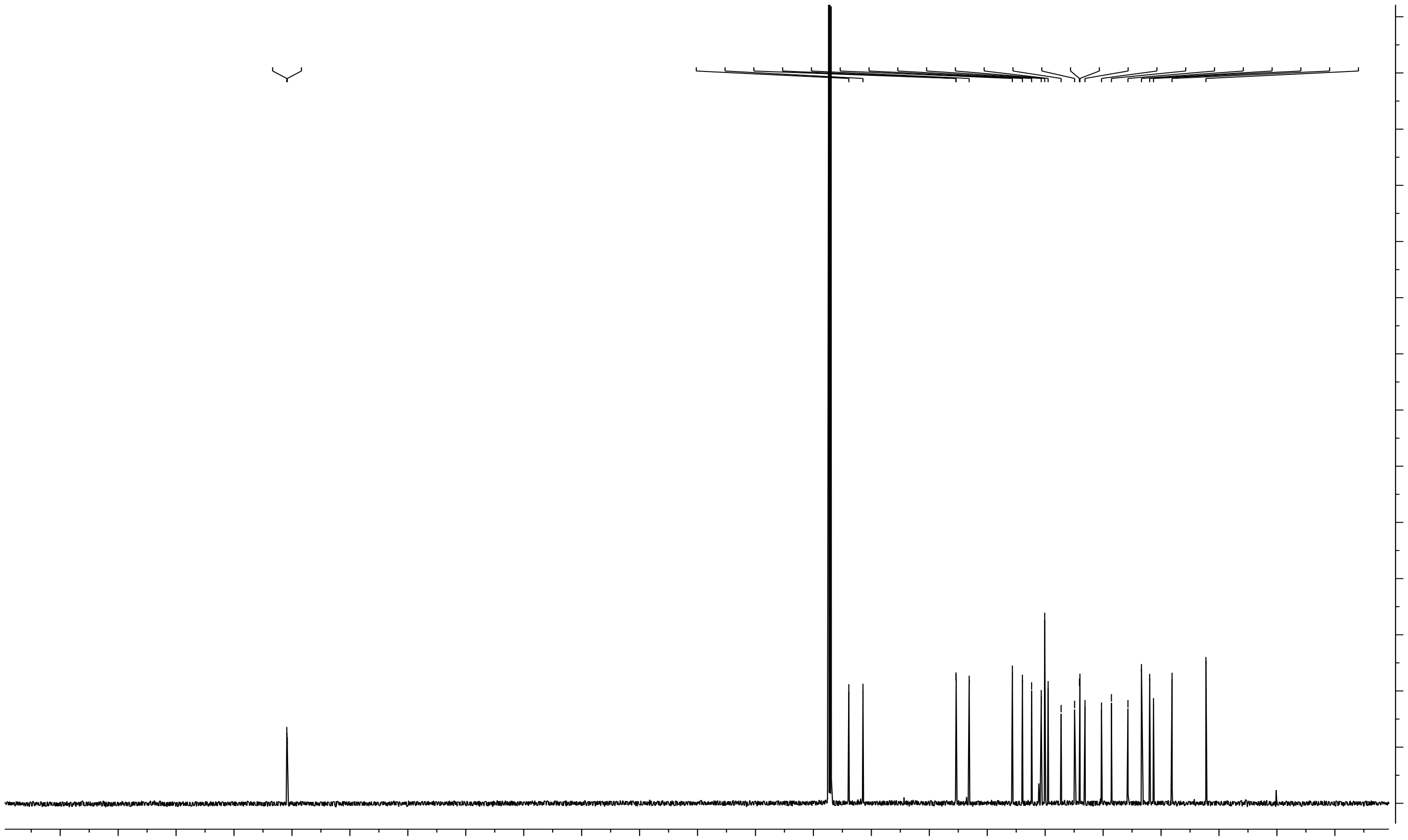

methyl (4R)-4-((3R,7S,10S,13R)-7-acetoxy-3-hydroxy-10,13-dimethylhexa-decahydr-o-1H-cyclopenta[a]phenanthren-17-yl)-2-bromopentanoate (MS) U12-Br:

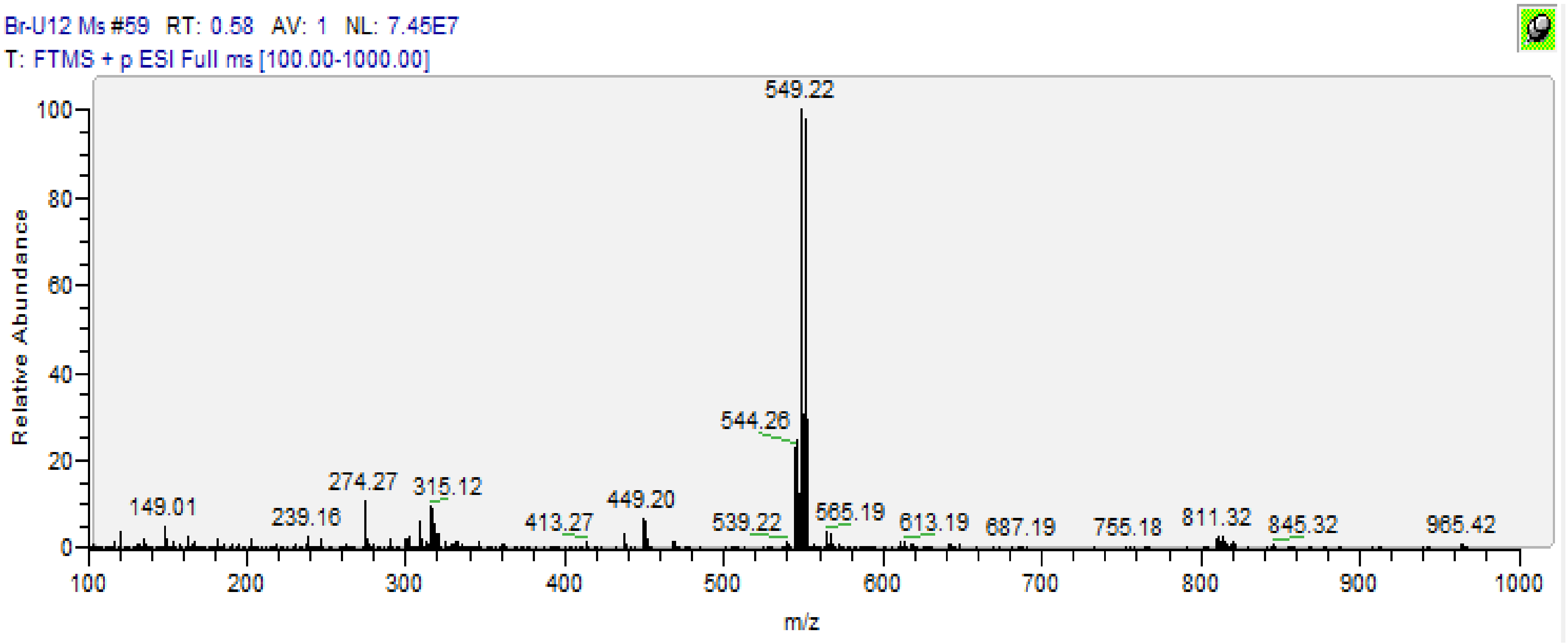

methyl (4R)-4-((3R,7S,10S,13R)-7-acetoxy-3-hydroxy-10,13-dimethylhexa-decahydr-o-1H-cyclopenta[a]phenanthren-17-yl)-2-iodopentanoate (^{1}H) U12-I:

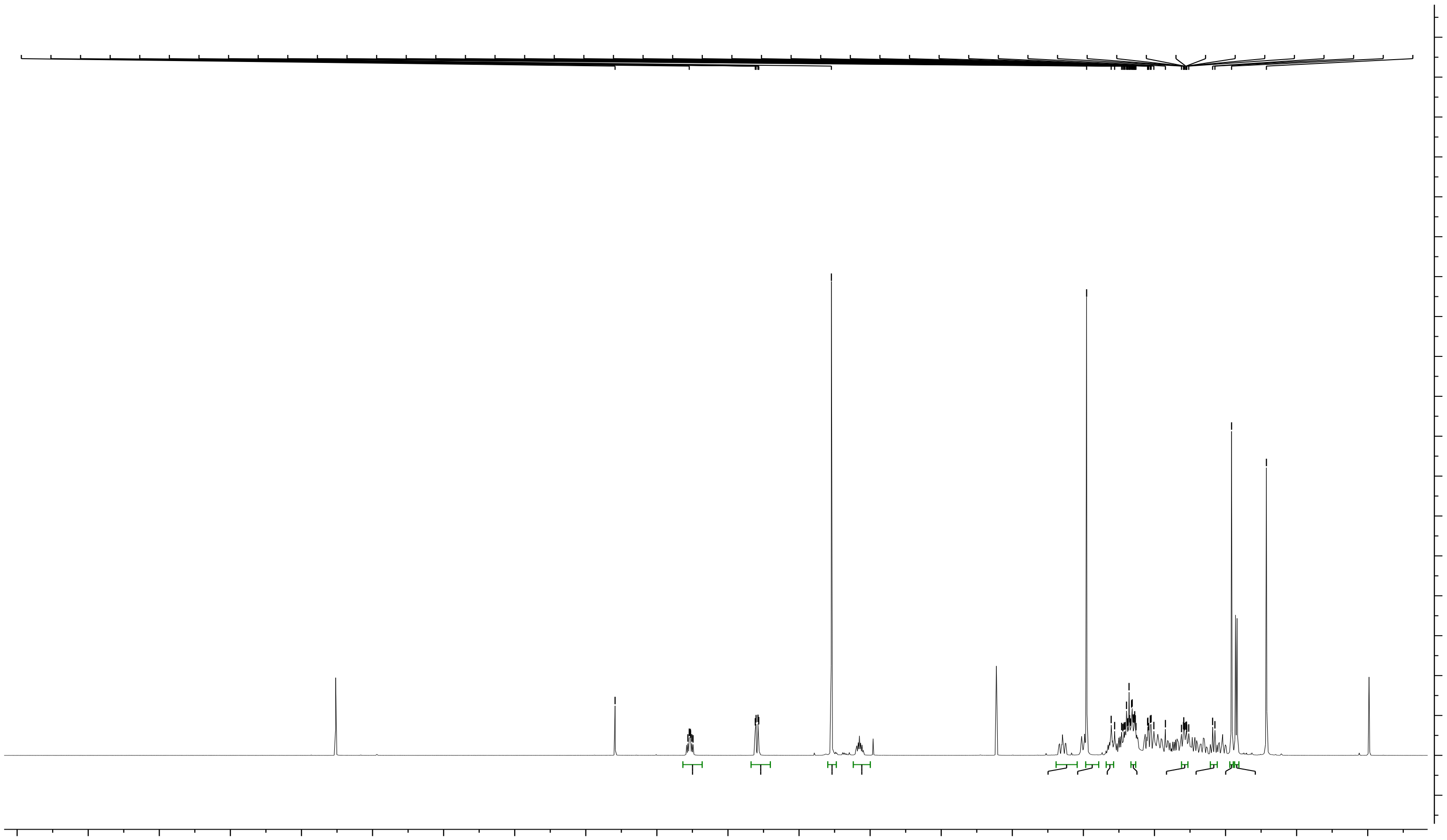

methyl (4R)-4-((3R,7S,10S,13R)-7-acetoxy-3-hydroxy-10,13-dimethylhexa-decahydr-o-1H-cyclopenta[a]phenanthren-17-yl)-2-iodopentanoate (^{13}C) U12-I:

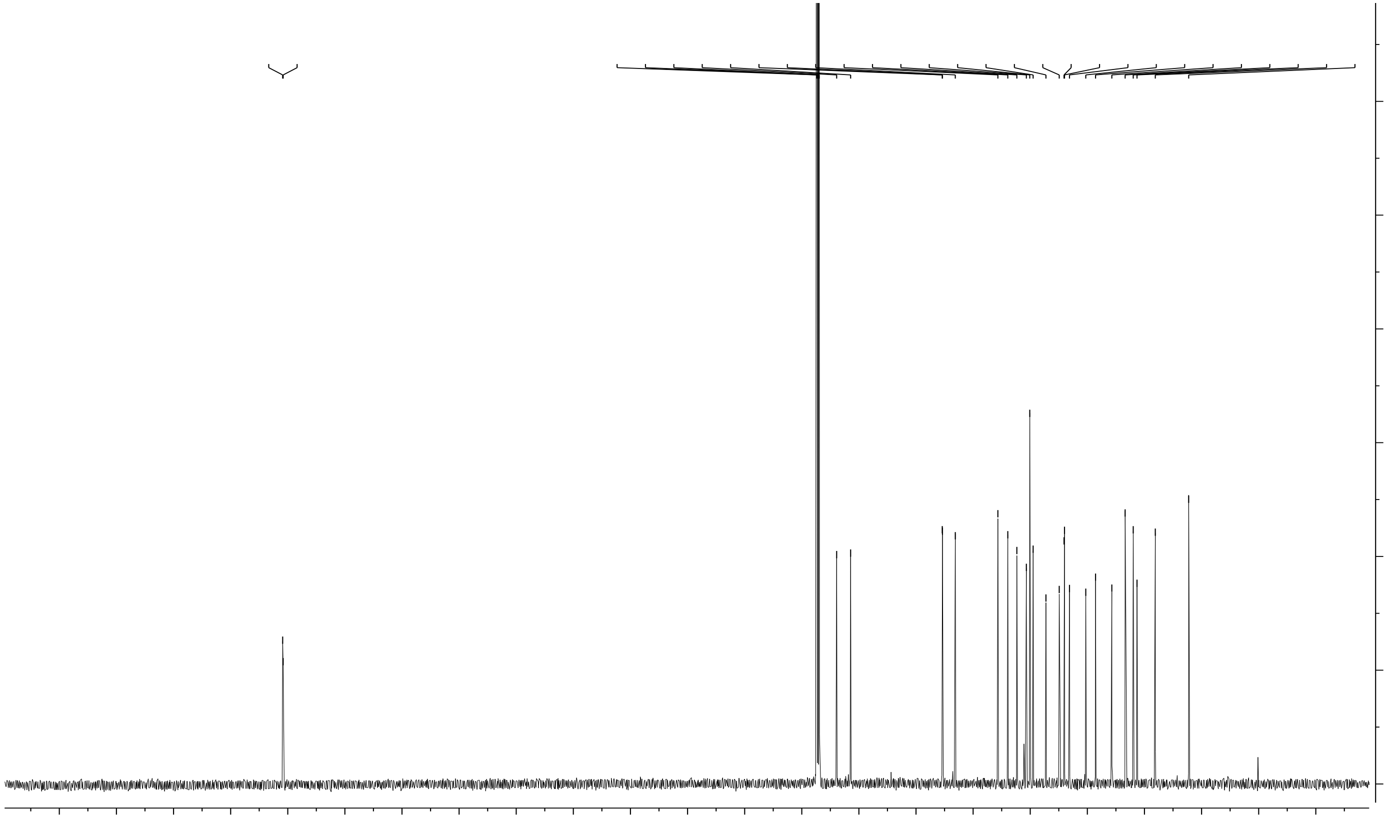

methyl (4R)-4-((3R,7S,10S,13R)-7-acetoxy-3-hydroxy-10,13-dimethylhexa-decahydr-o-1H-cyclopenta[a]phenanthren-17-yl)-2-iodopentanoate (MS) U12-I:

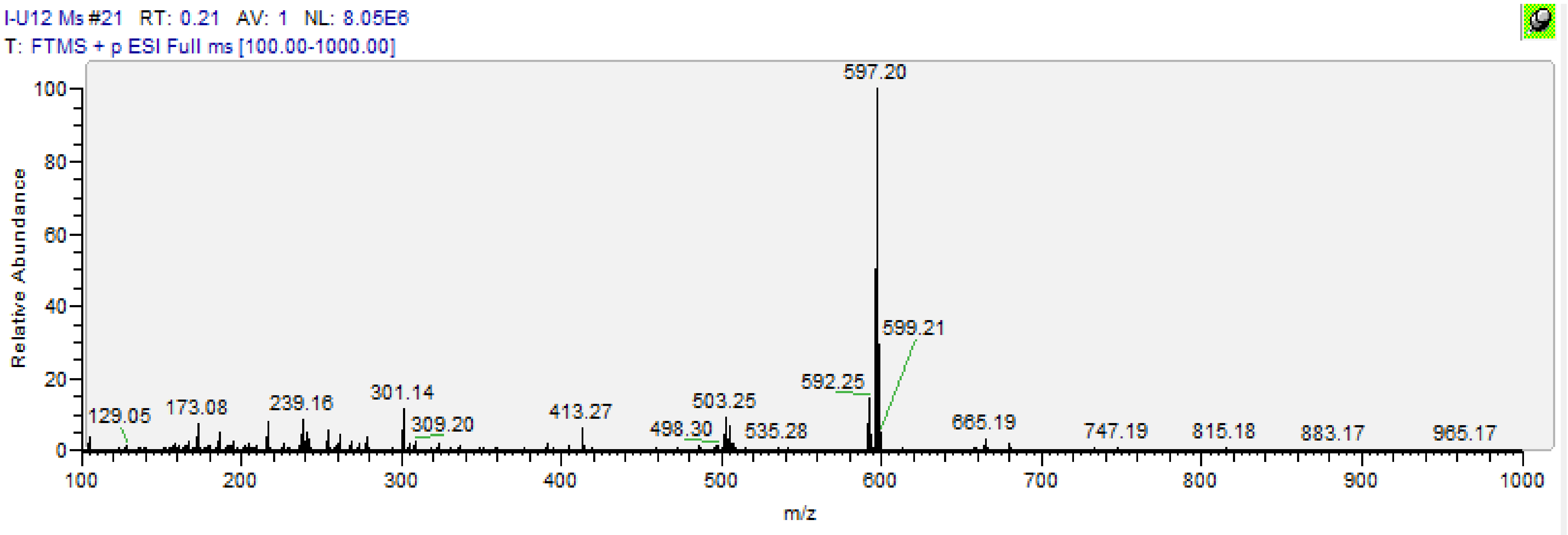

(3R,7S,10S,13R)-17-((R)-5-(4-benzylpiperazin-1-yl)-5-oxopentan-2-yl)-3-hyd-roxy-10,13-dimethylhexadecahydro-1H-cyclopenta[a]phenanthren-7-yl acetate (^{1}H) U12a:

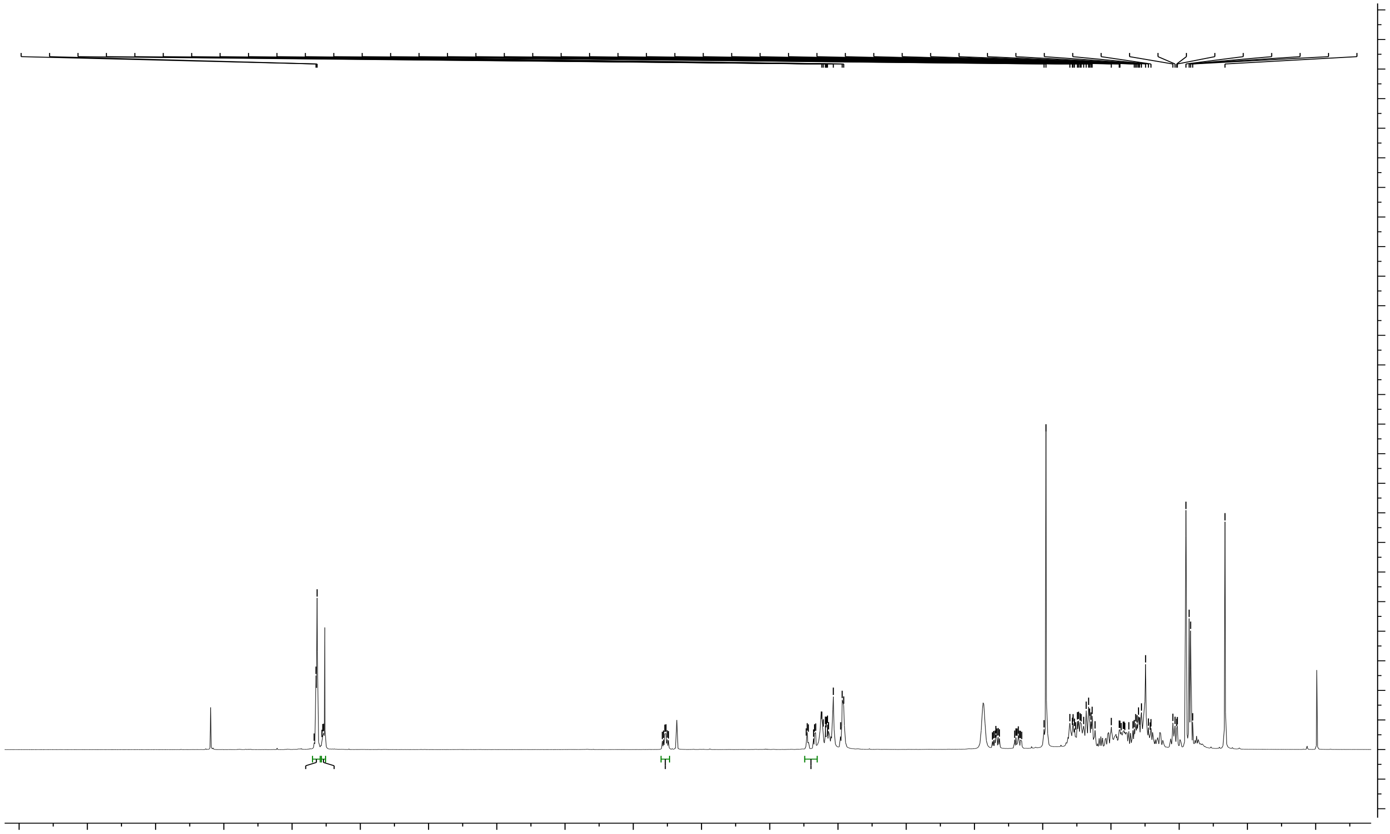

(3R,7S,10S,13R)-17-((R)-5-(4-benzylpiperazin-1-yl)-5-oxopentan-2-yl)-3-hyd-roxy-10,13-dimethylhexadecahydro-1H-cyclopenta[a]phenanthren-7-yl acetate (^{13}C) U12a:

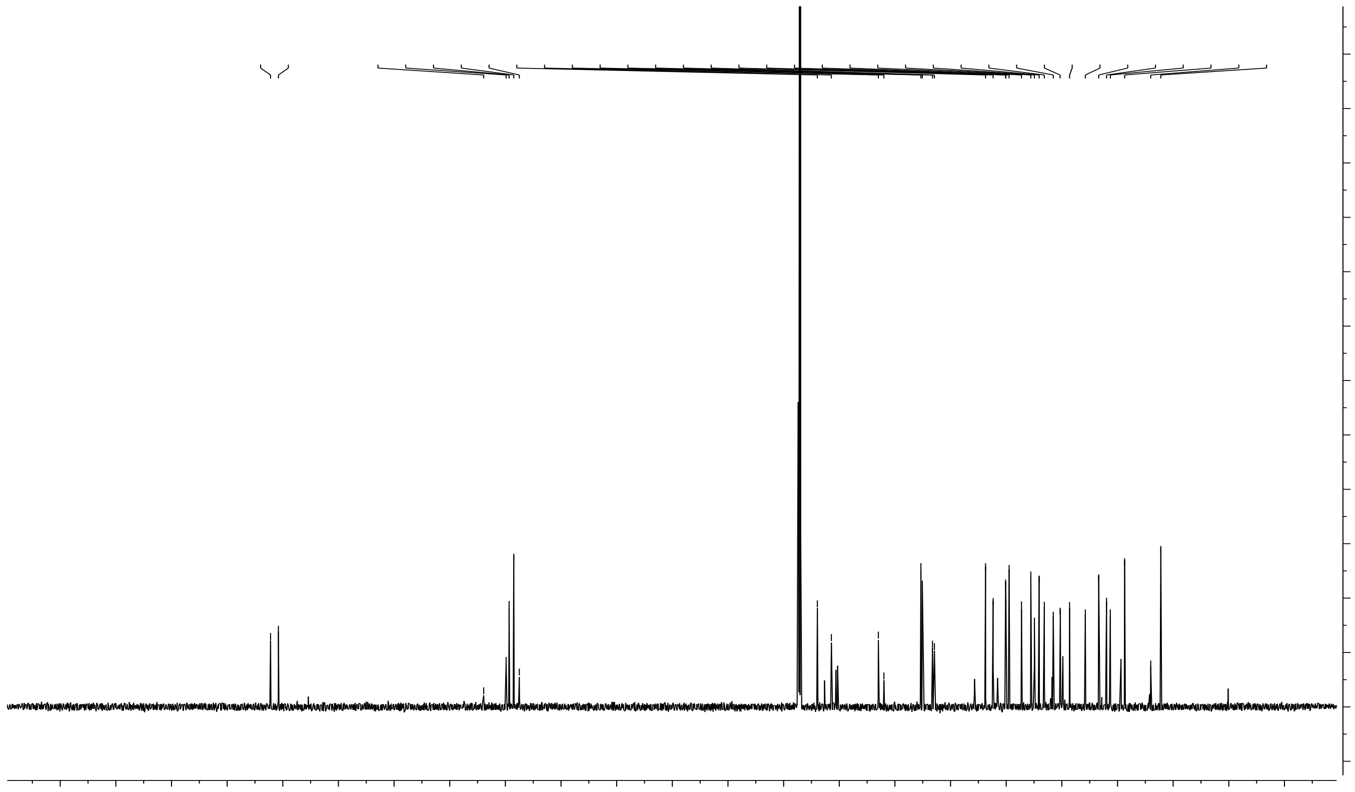

(3R,7S,10S,13R)-17-((R)-5-(4-benzylpiperazin-1-yl)-5-oxopentan-2-yl)-3-hyd-roxy-10,13-dimethylhexadecahydro-1H-cyclopenta[a]phenanthren-7-yl acetate (MS) U12a:

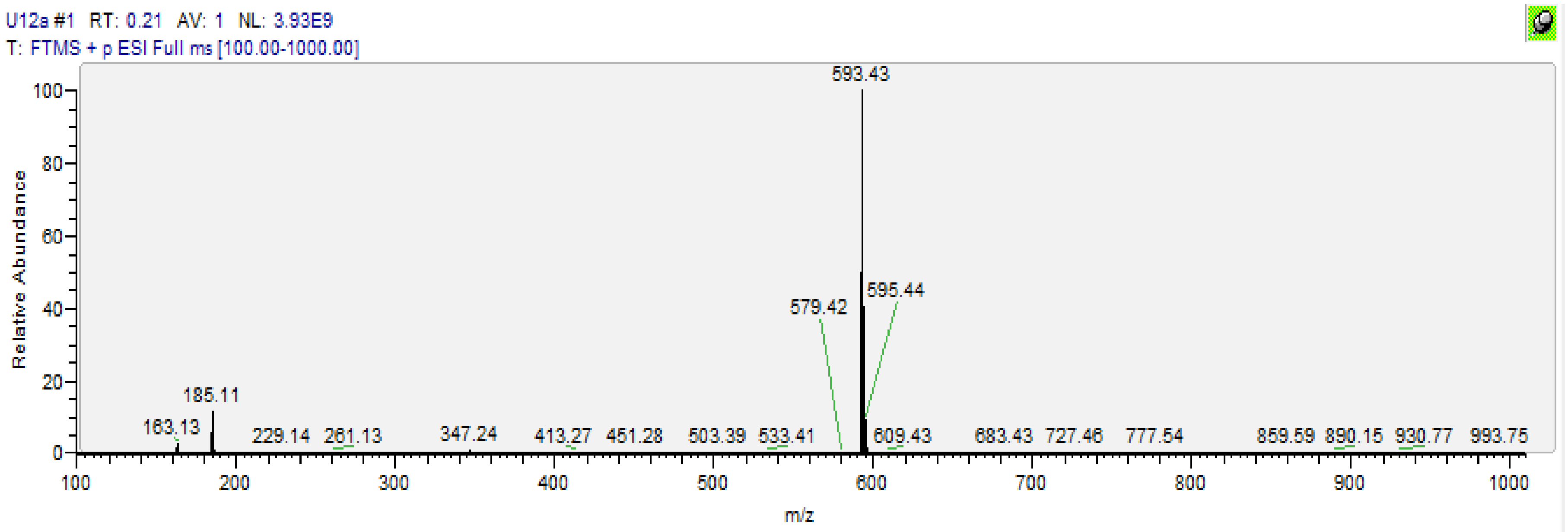

(3R,7S,10S,13R)-17-((R)-5-(4-cinnamylpiperazin-1-yl)-5-oxopentan-2-yl)-3-hydroxy--10,13-dimethylhexadecahydro-1H-cyclopenta[a]phenanthren-7-yl acetate (^{1}H) U12b:

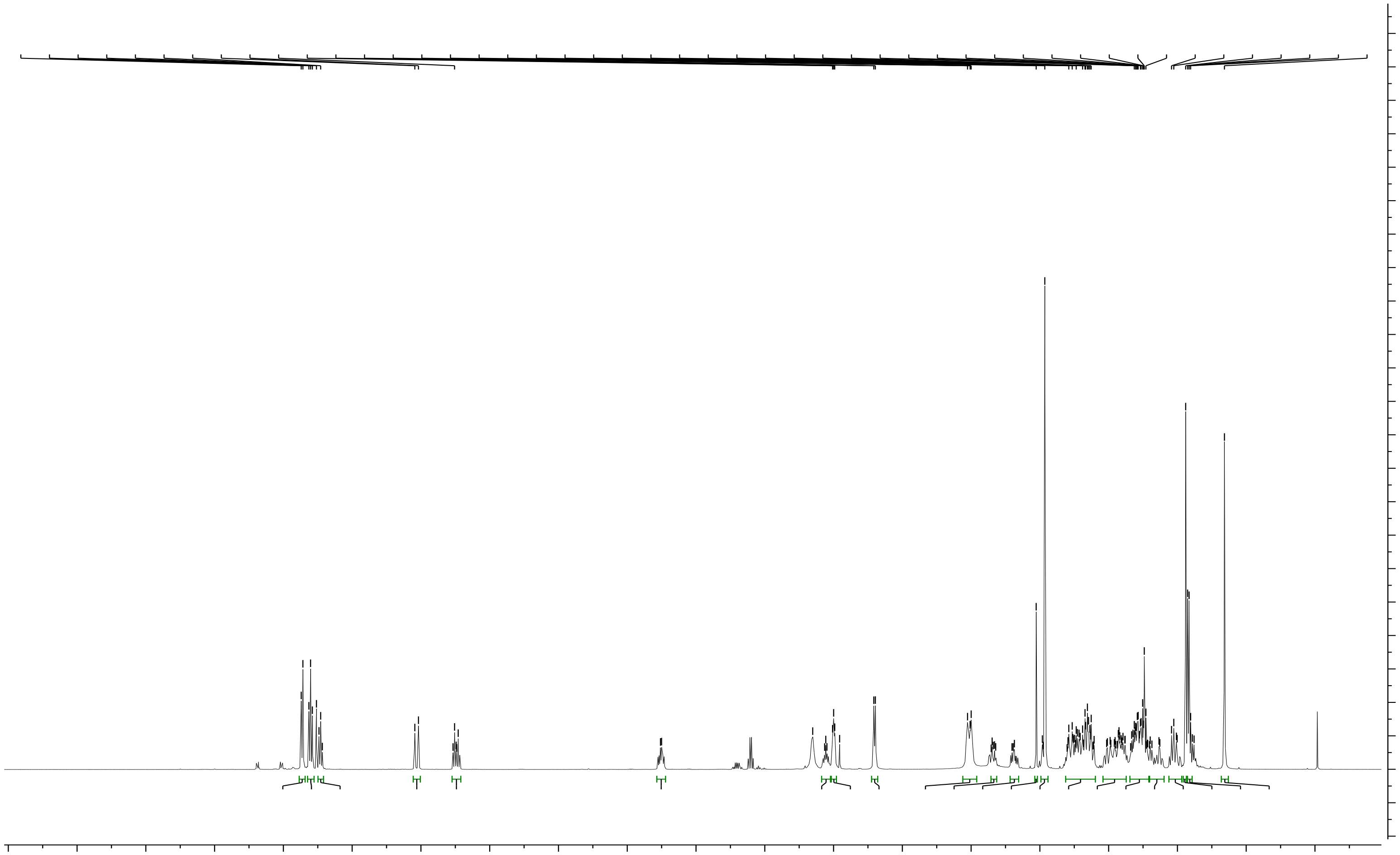

(3R,7S,10S,13R)-17-((R)-5-(4-cinnamylpiperazin-1-yl)-5-oxopentan-2-yl)-3-hydroxy--10,13-dimethylhexadecahydro-1H-cyclopenta[a]phenanthren-7-yl acetate (^{13}C) U12b:

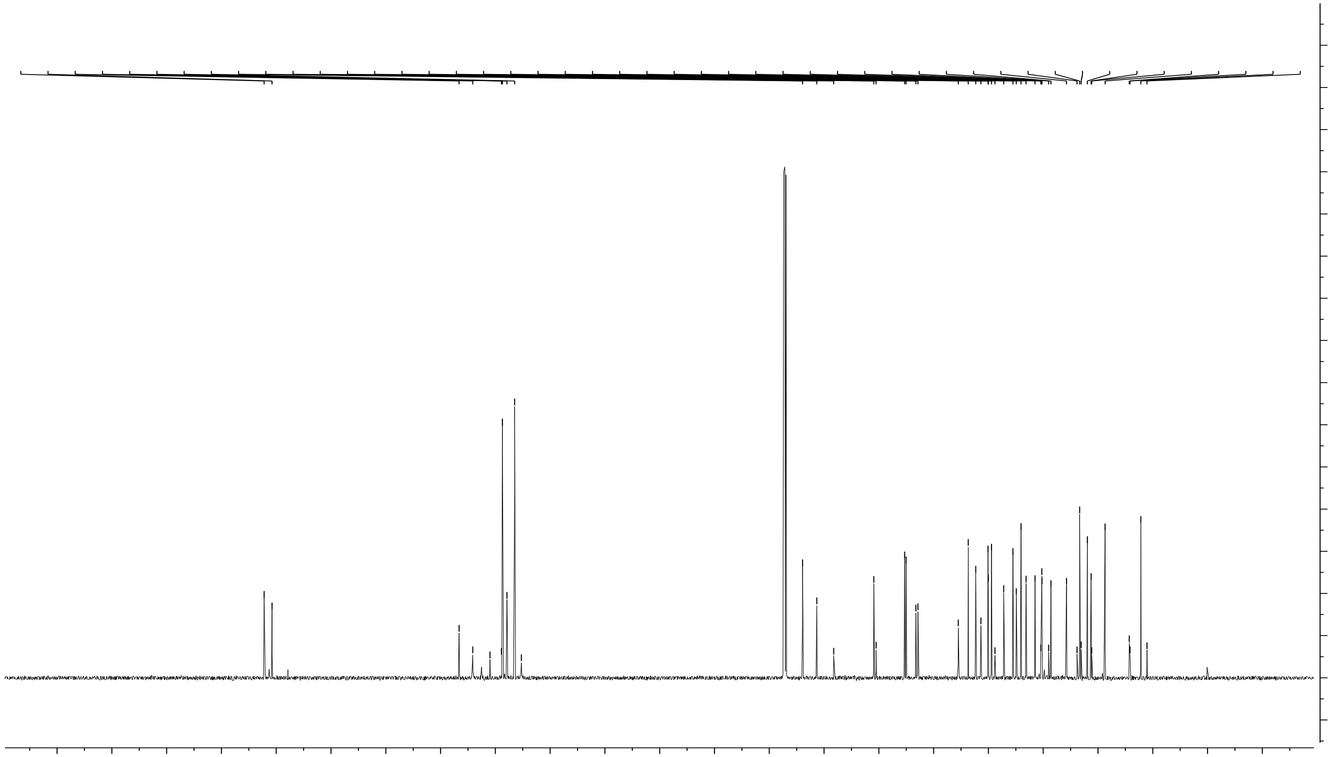

(3R,7S,10S,13R)-17-((R)-5-(4-cinnamylpiperazin-1-yl)-5-oxopentan-2-yl)-3-hydroxy--10,13-dimethylhexadecahydro-1H-cyclopenta[a]phenanthren-7-yl acetate (MS) U12b:

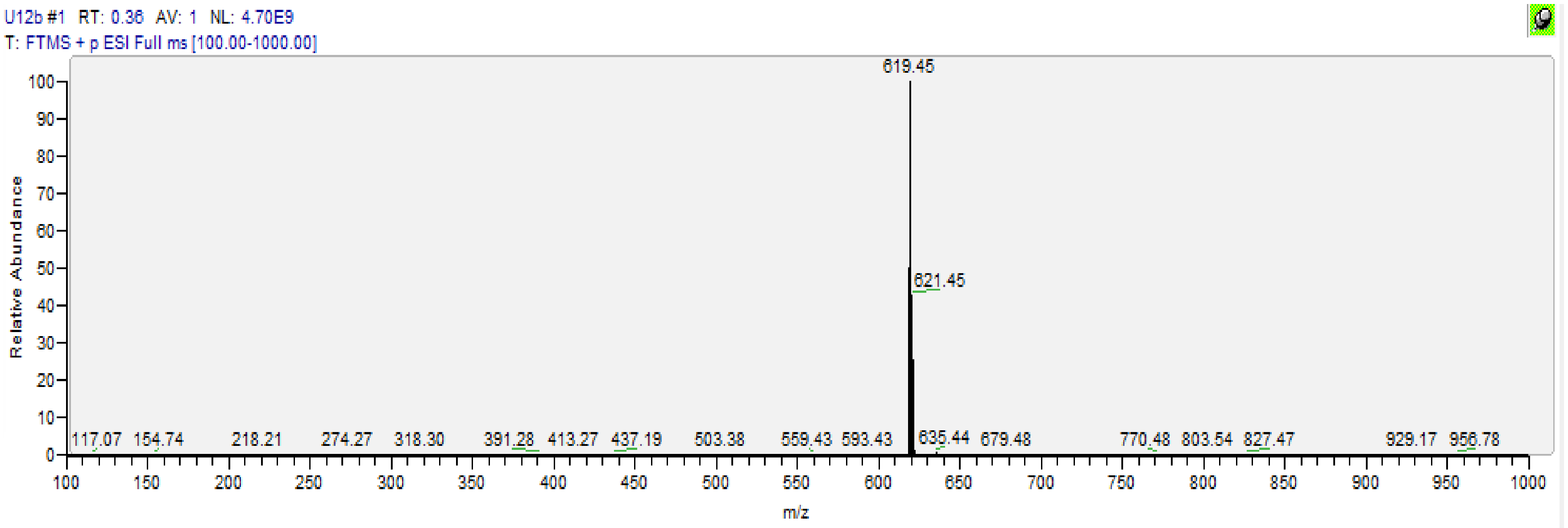

(3R,7S,10S,13R)-3-hydroxy-10,13-dimethyl-17-((R)-5-oxo-5- (piperazin-1-yl) pentan-2-yl) hexadecahydro-1H-cyclopenta[a]phenanthren-7-yl acetate (^{1}H) U12c:

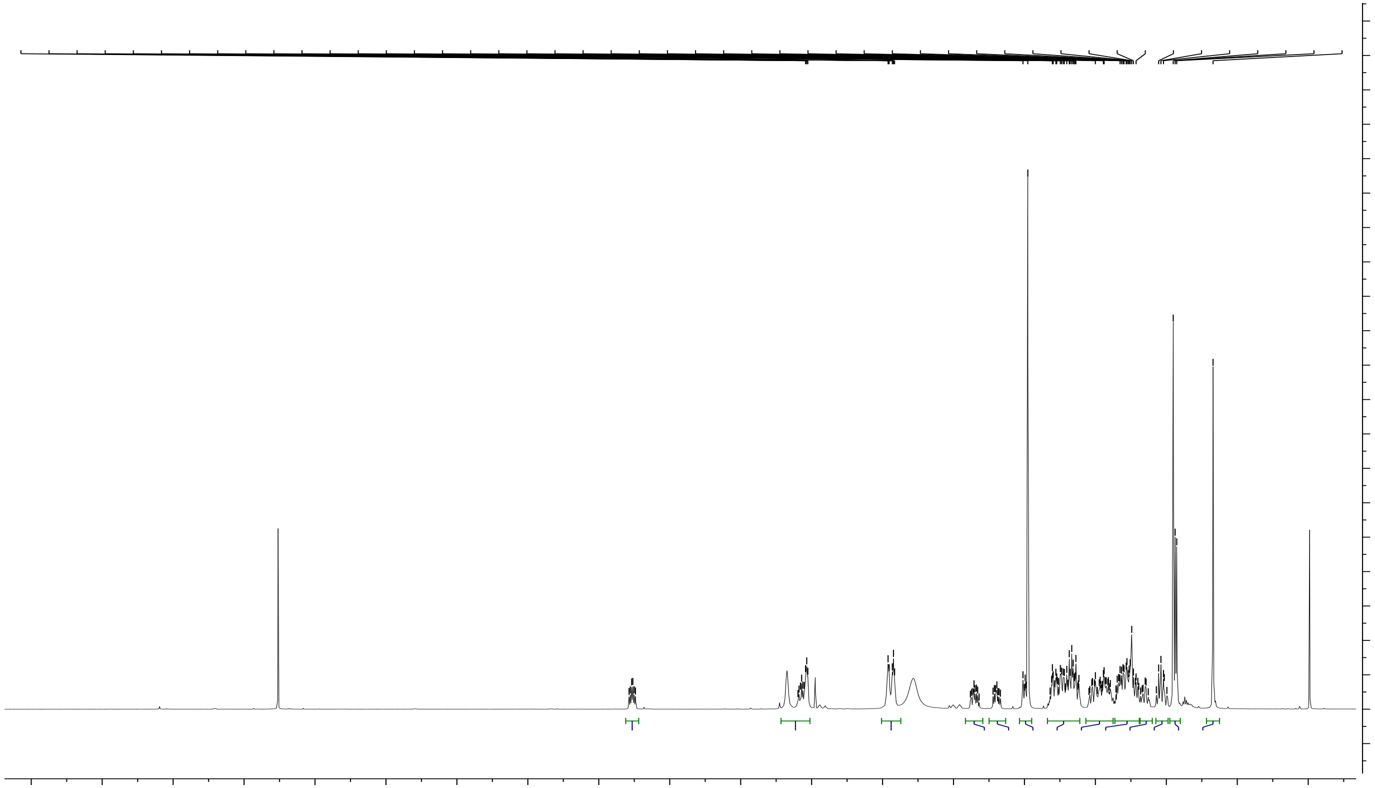

(3R,7S,10S,13R)-3-hydroxy-10,13-dimethyl-17-((R)-5-oxo-5- (piperazin-1-yl) pentan-2-yl) hexadecahydro-1H-cyclopenta[a]phenanthren-7-yl acetate (^{13}C) U12c:

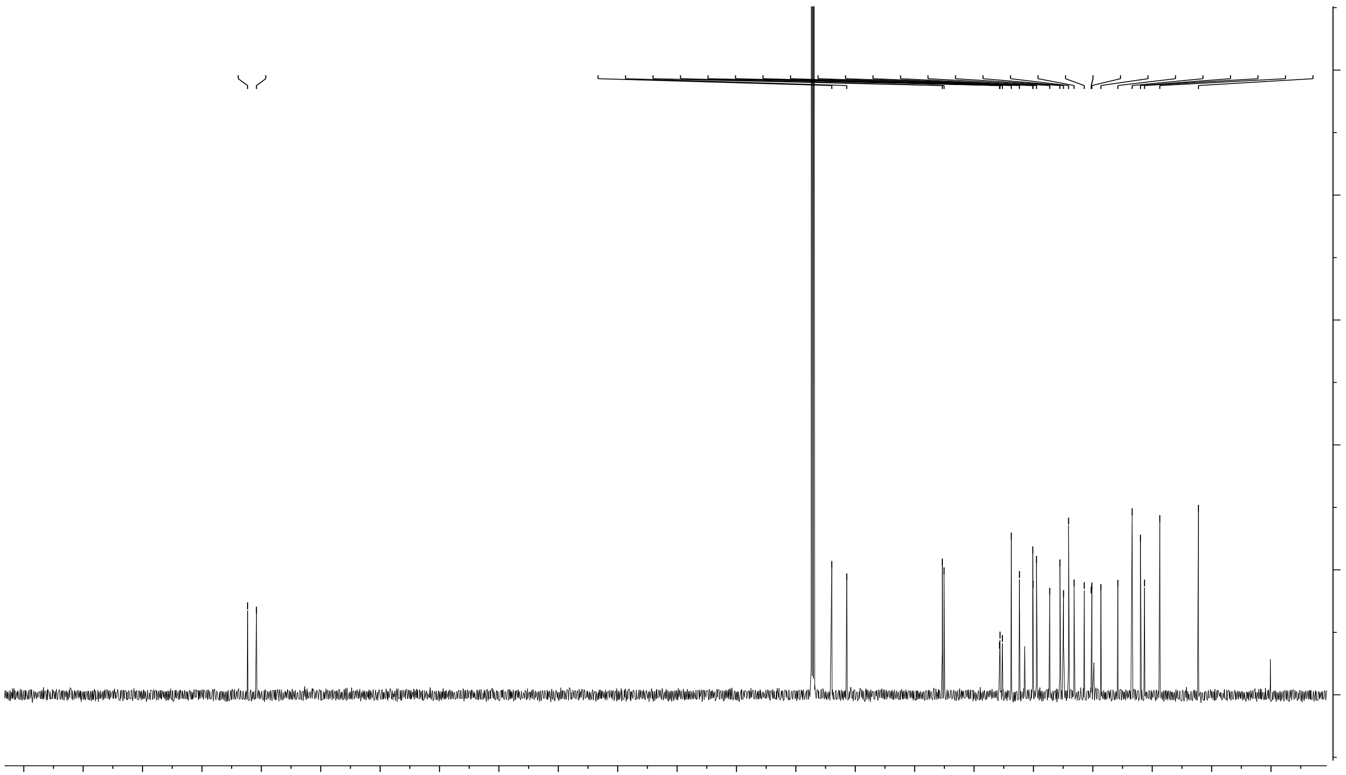

(3R,7S,10S,13R)-3-hydroxy-10,13-dimethyl-17-((R)-5-oxo-5- (piperazin-1-yl) pentan-2-yl) hexadecahydro-1H-cyclopenta[a]phenanthren-7-yl acetate (MS) U12c:

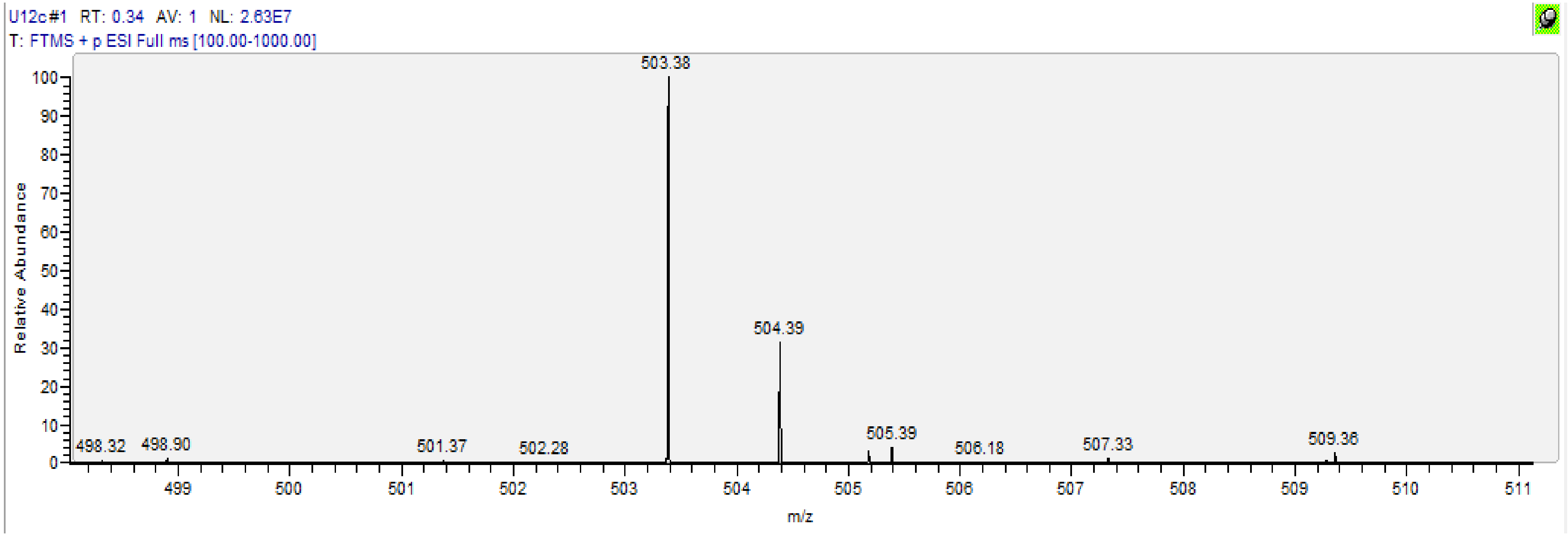

(3R,7S,10S,13R)-17-((R)-5-(cyclopropylamino)-5-oxopentan-2-yl) -3-hydro-xy-10,13-dimethylhexadecahydro-1H-cyclopenta[a]phenanthren-7-yl acetate (^{1}H) U12d:

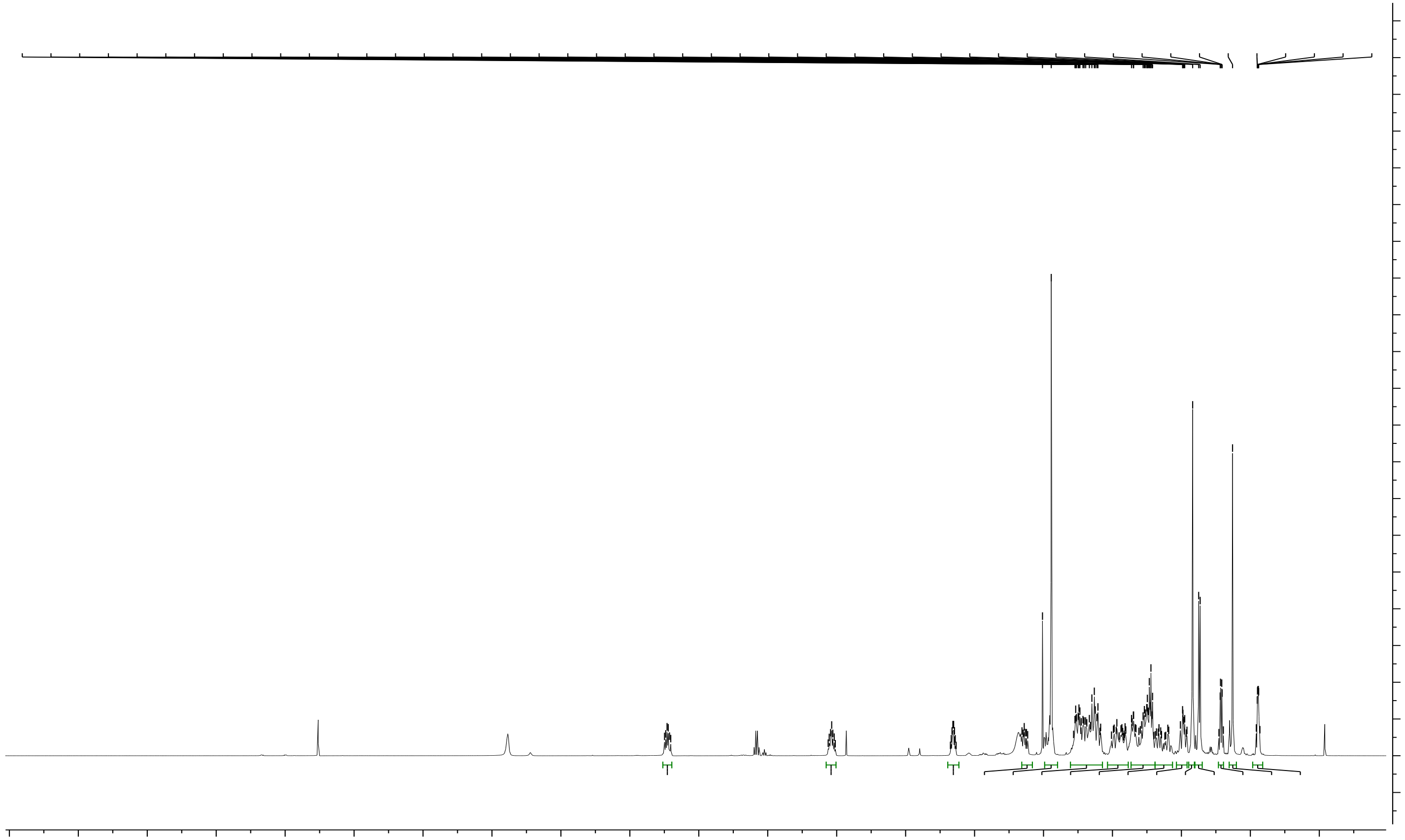

(3R,7S,10S,13R)-17-((R)-5-(cyclopropylamino)-5-oxopentan-2-yl) -3-hydro-xy-10,13-dimethylhexadecahydro-1H-cyclopenta[a]phenanthren-7-yl acetate (^{13}C) U12d:

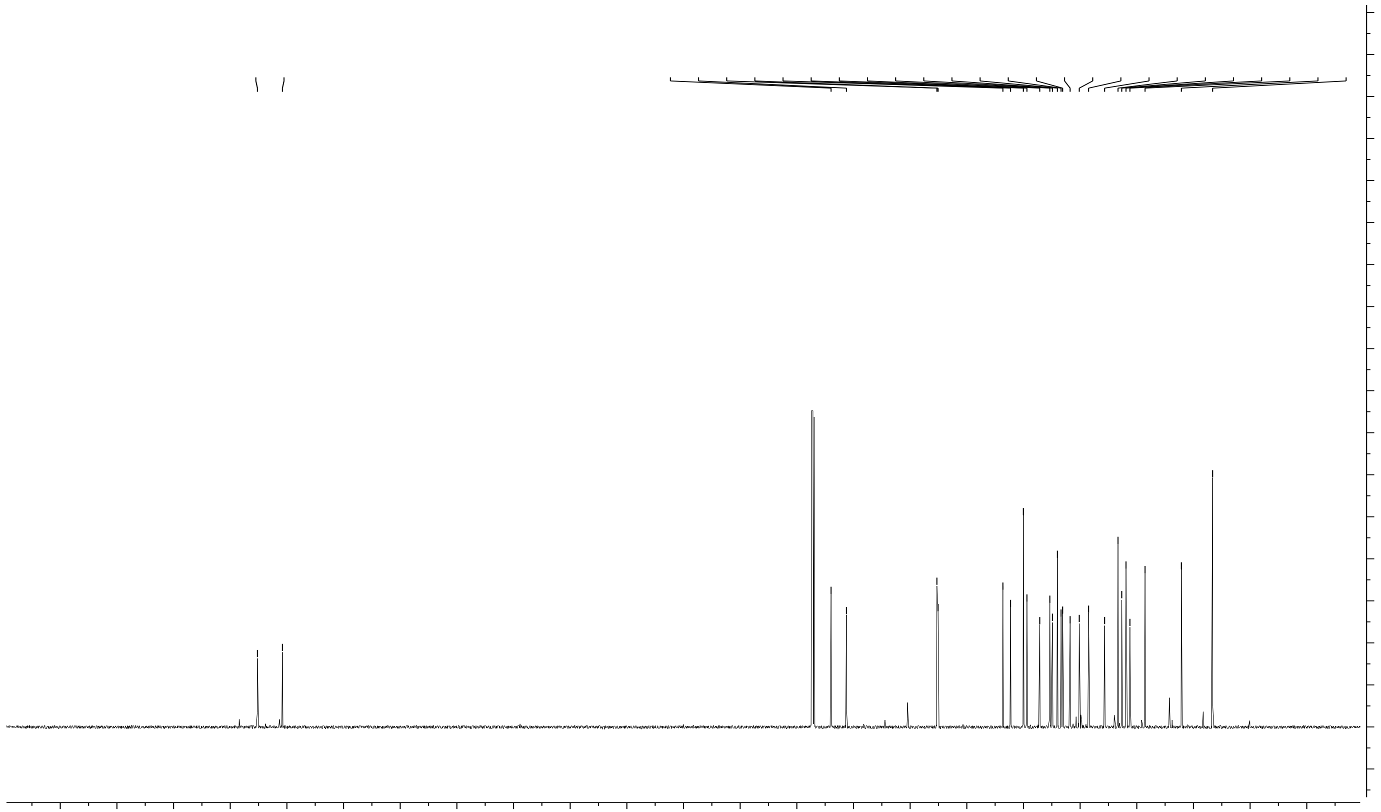

(3R,7S,10S,13R)-17-((R)-5-(cyclopropylamino)-5-oxopentan-2-yl) -3-hydro-xy-10,13-dimethylhexadecahydro-1H-cyclopenta[a]phenanthren-7-yl acetate (MS) U12d:

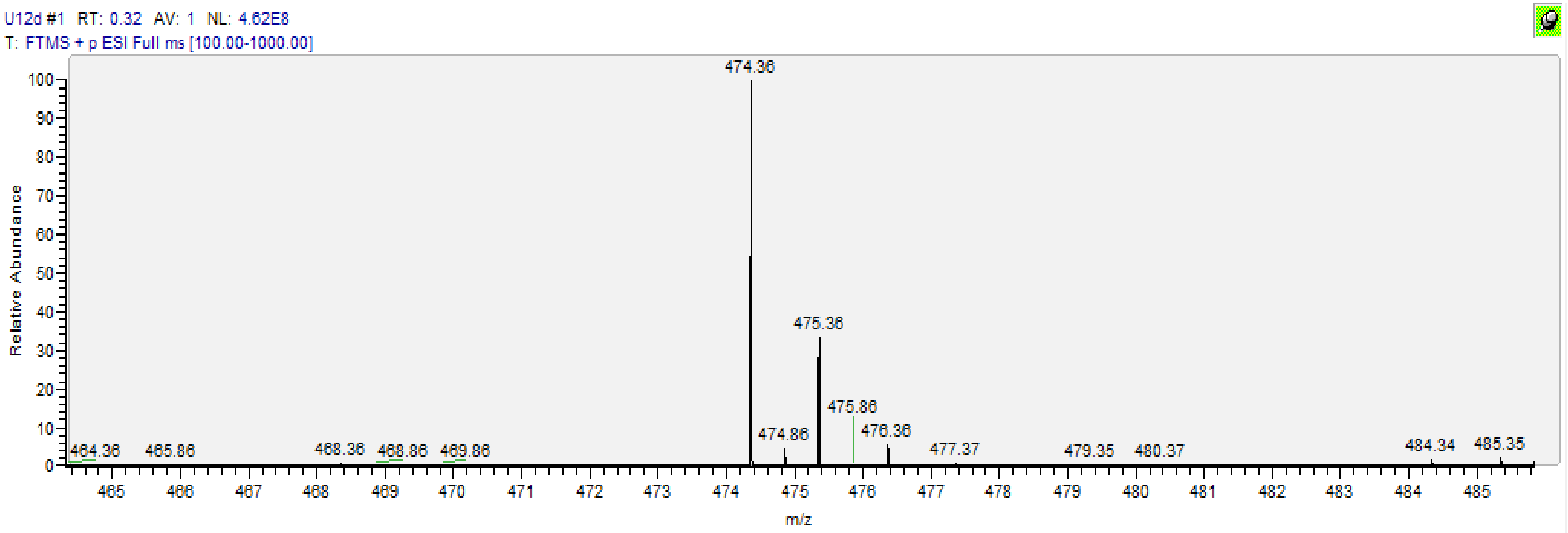

(4R)-4-((3R,7S,10S,13R)-3,7-dihydroxy-10,13- dimethylhexadecahydro-1H-cyclopenta[a]phenanthren-17-yl)-N-(4-methylpiperazin-1-yl) pentanamide (^{1}H) U12e:

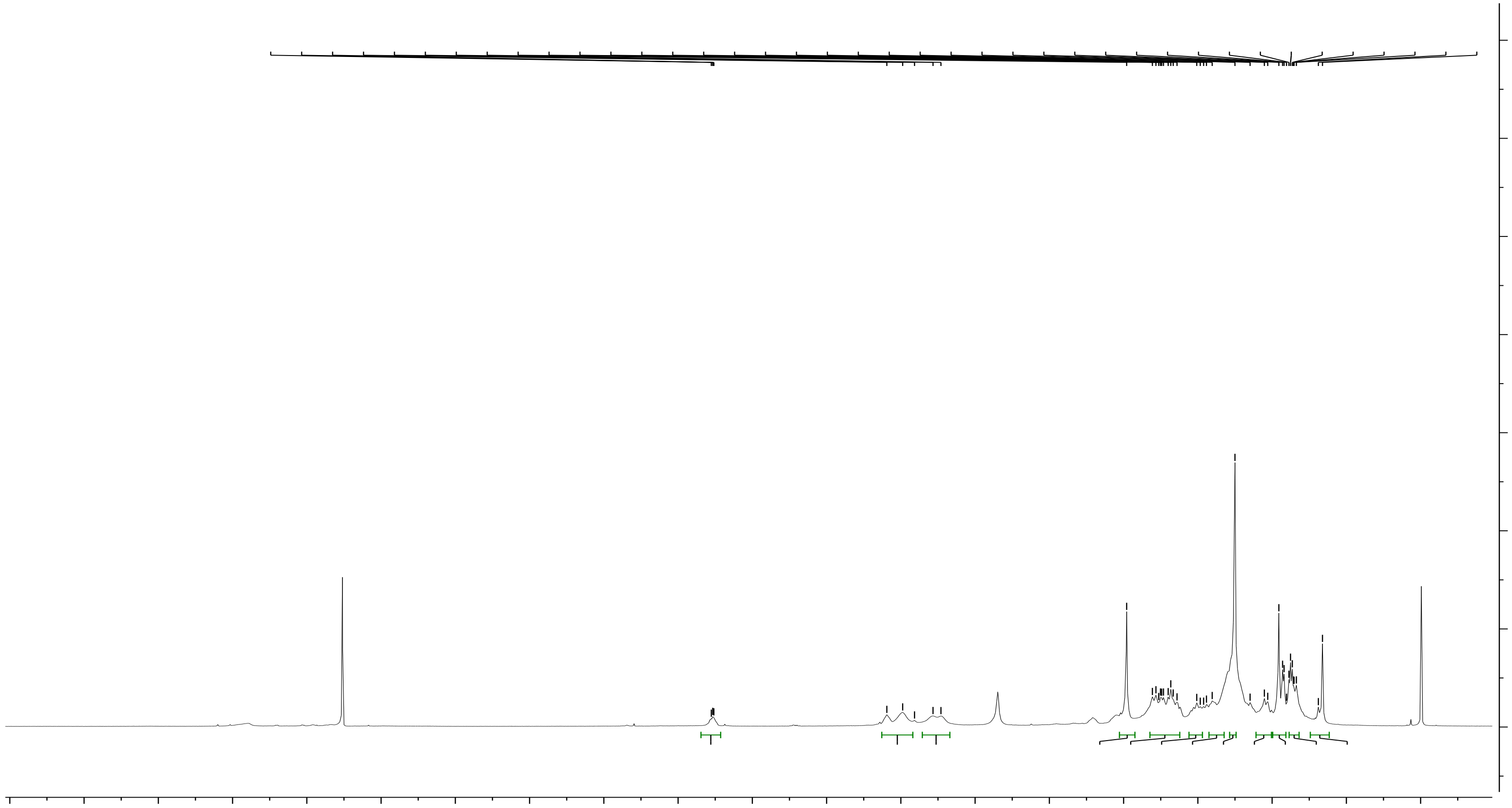

(4R)-4-((3R,7S,10S,13R)-3,7-dihydroxy-10,13- dimethylhexadecahydro-1H-cyclopenta[a]phenanthren-17-yl)-N-(4-methylpiperazin-1-yl) pentanamide (^{13}C) U12e:

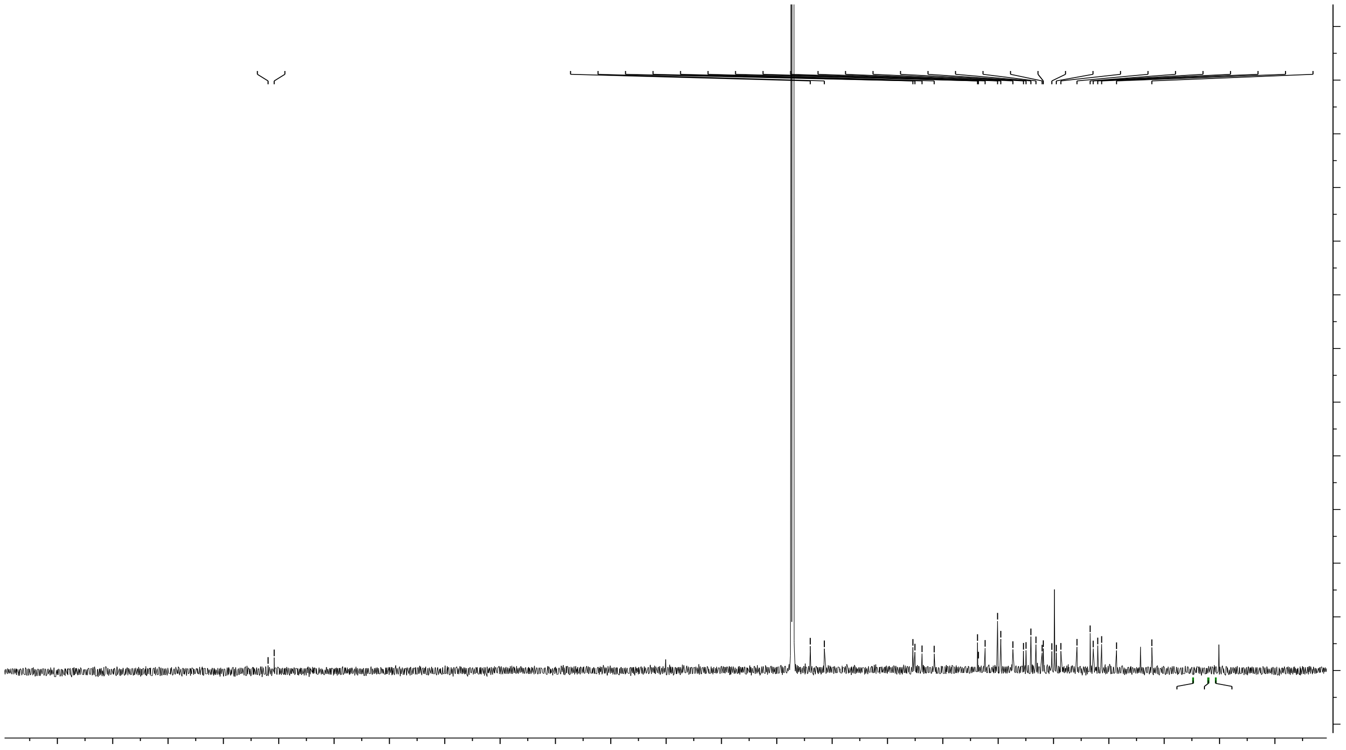

(4R)-4-((3R,7S,10S,13R)-3,7-dihydroxy-10,13- dimethylhexadecahydro-1H-cyclopenta[a]phenanthren-17-yl)-N-(4-methylpiperazin-1-yl) pentanamide (MS) U12e:

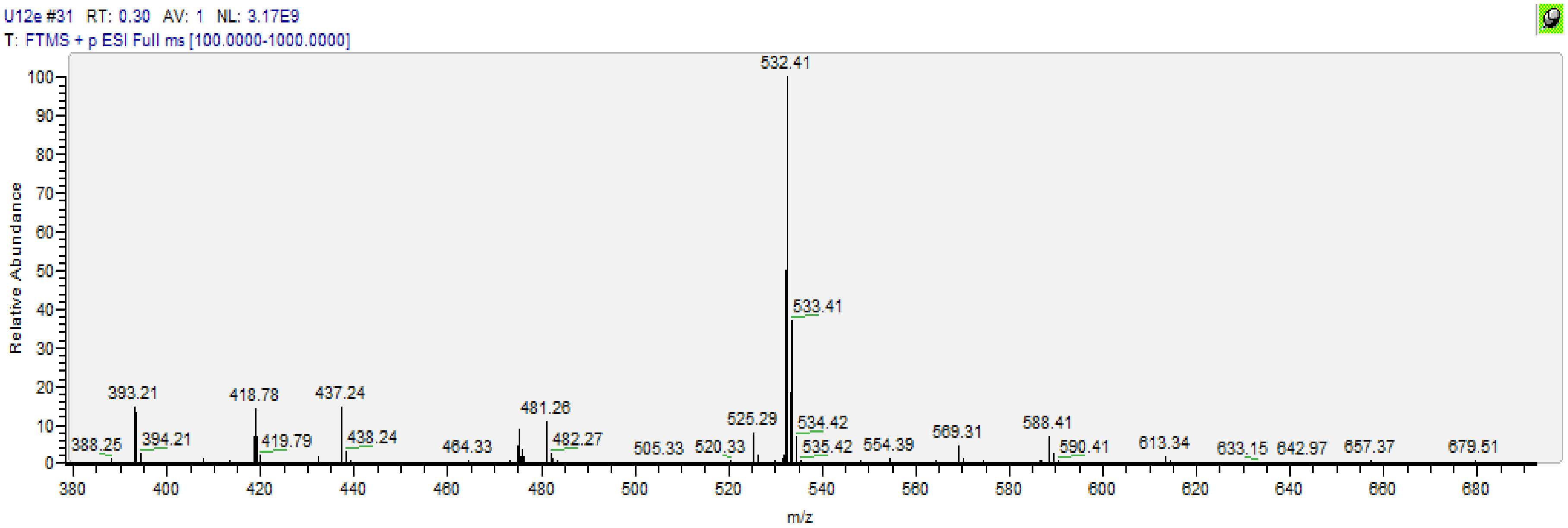

(3R,7S,10S,13R)-3-hydroxy-10,13-dimethyl-17-((R)-5-oxo-5- (p-tolyl amino) pentan-2-yl) hexadecahydro-1H-cyclopenta[a]phenanthren-7-yl acetate (^{1}H) U12f:

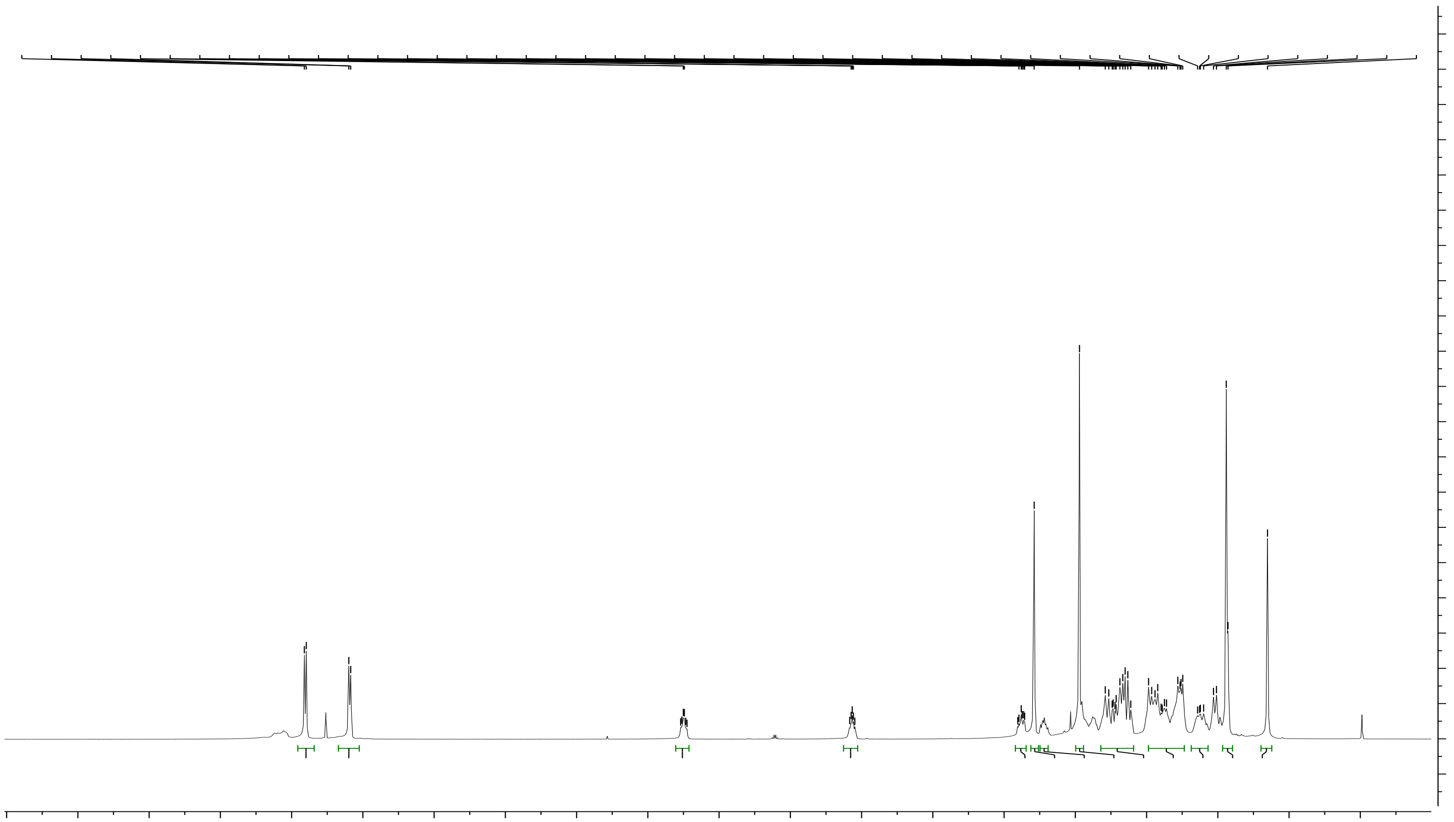

(3R,7S,10S,13R)-3-hydroxy-10,13-dimethyl-17-((R)-5-oxo-5- (p-tolyl amino) pentan-2-yl) hexadecahydro-1H-cyclopenta[a]phenanthren-7-yl acetate (^{13}C) U12f:

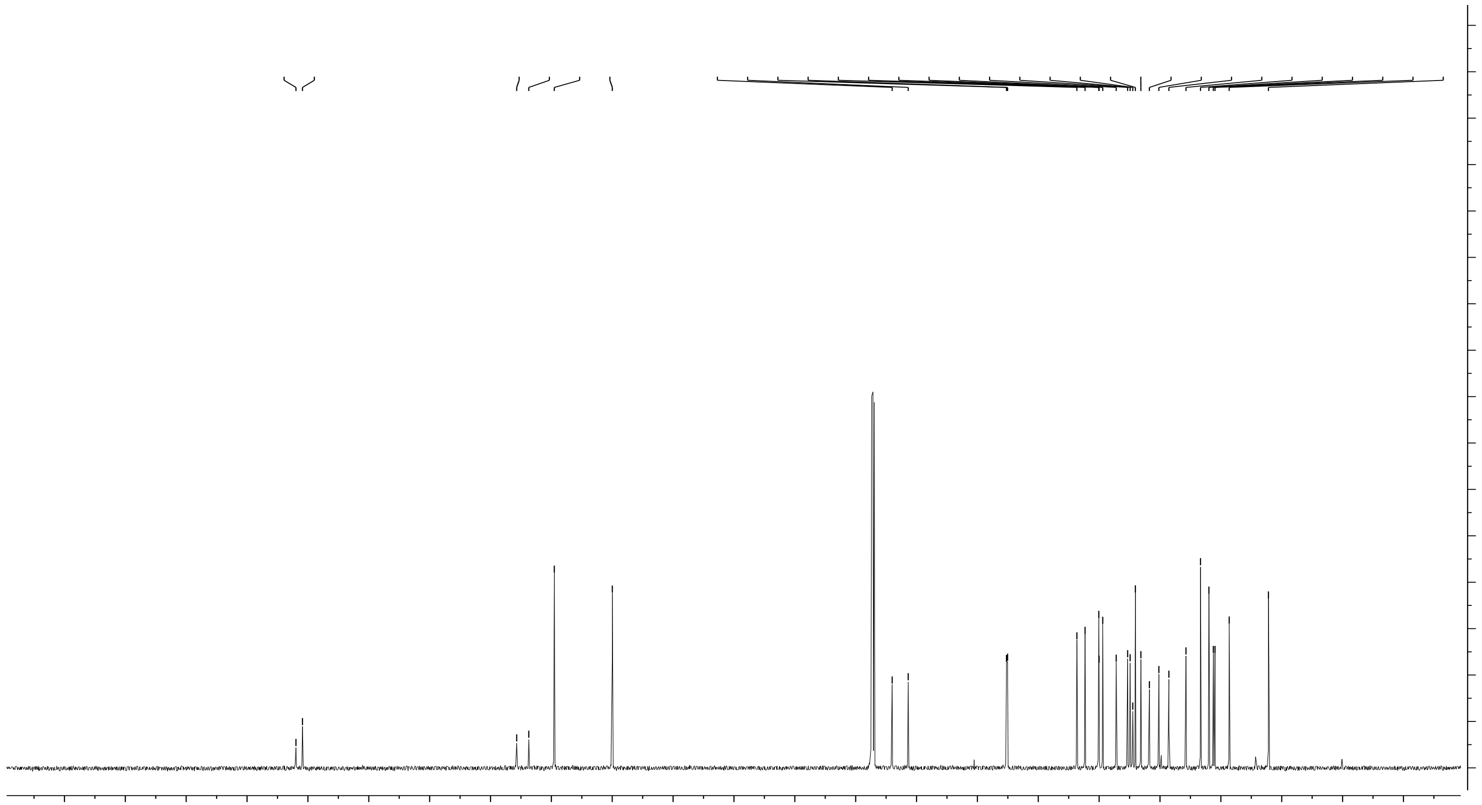

(3R,7S,10S,13R)-3-hydroxy-10,13-dimethyl-17-((R)-5-oxo-5- (p-tolyl amino) pentan-2-yl) hexadecahydro-1H-cyclopenta[a]phenanthren-7-yl acetate (MS) U12f:

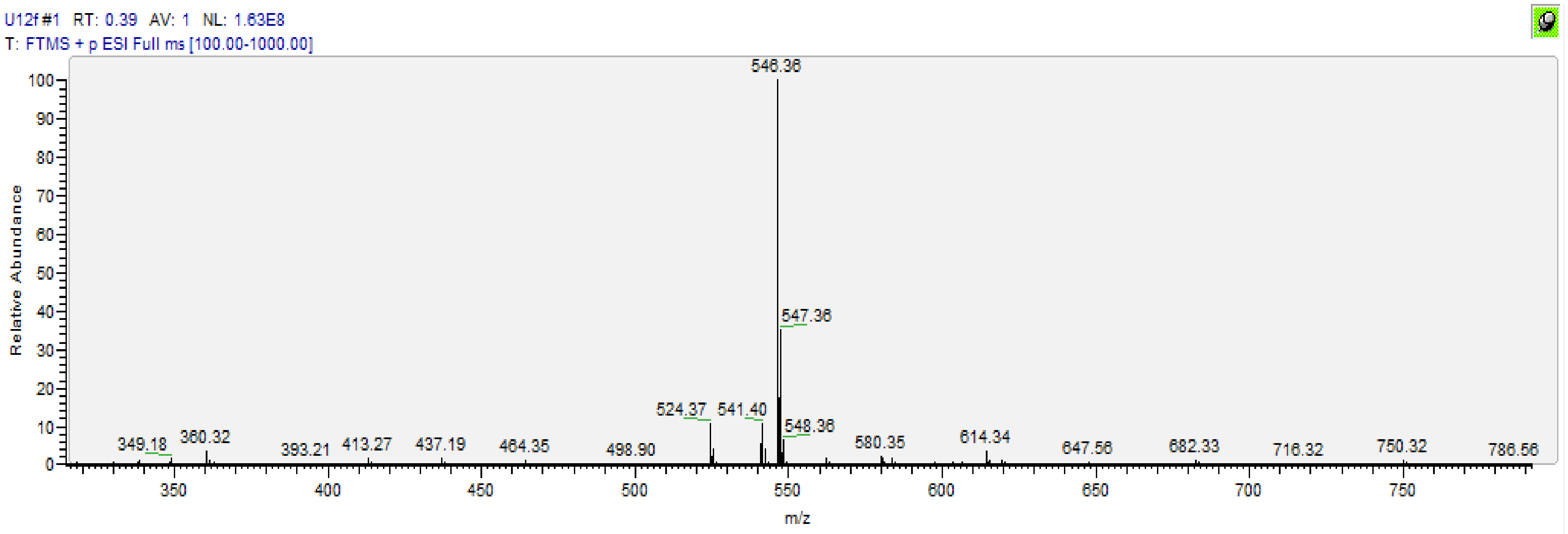

(3R,7S,10S,13R)-17-((R)-5-((4-chlorophenyl) amino)-5-oxopentan-2-yl)-3-h-ydroxy-10,13-dimethylhexadecahydro-1H-cyclopenta[a]phenanthren-7-yl acetate (^{1}H) U12g:

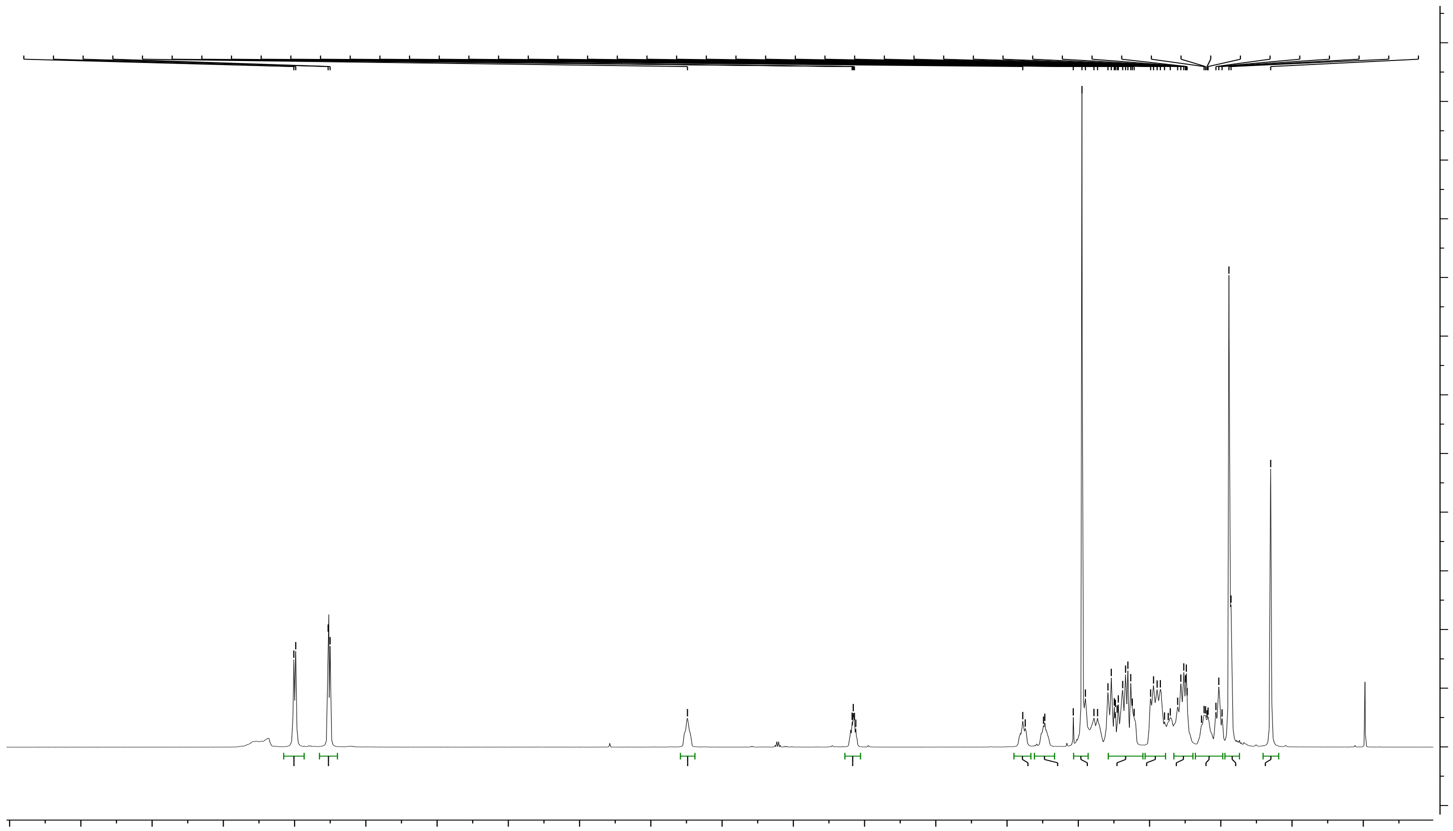

(3R,7S,10S,13R)-17-((R)-5-((4-chlorophenyl) amino)-5-oxopentan-2-yl)-3-h-ydroxy-10,13-dimethylhexadecahydro-1H-cyclopenta[a]phenanthren-7-yl acetate (^{13}C) U12g:

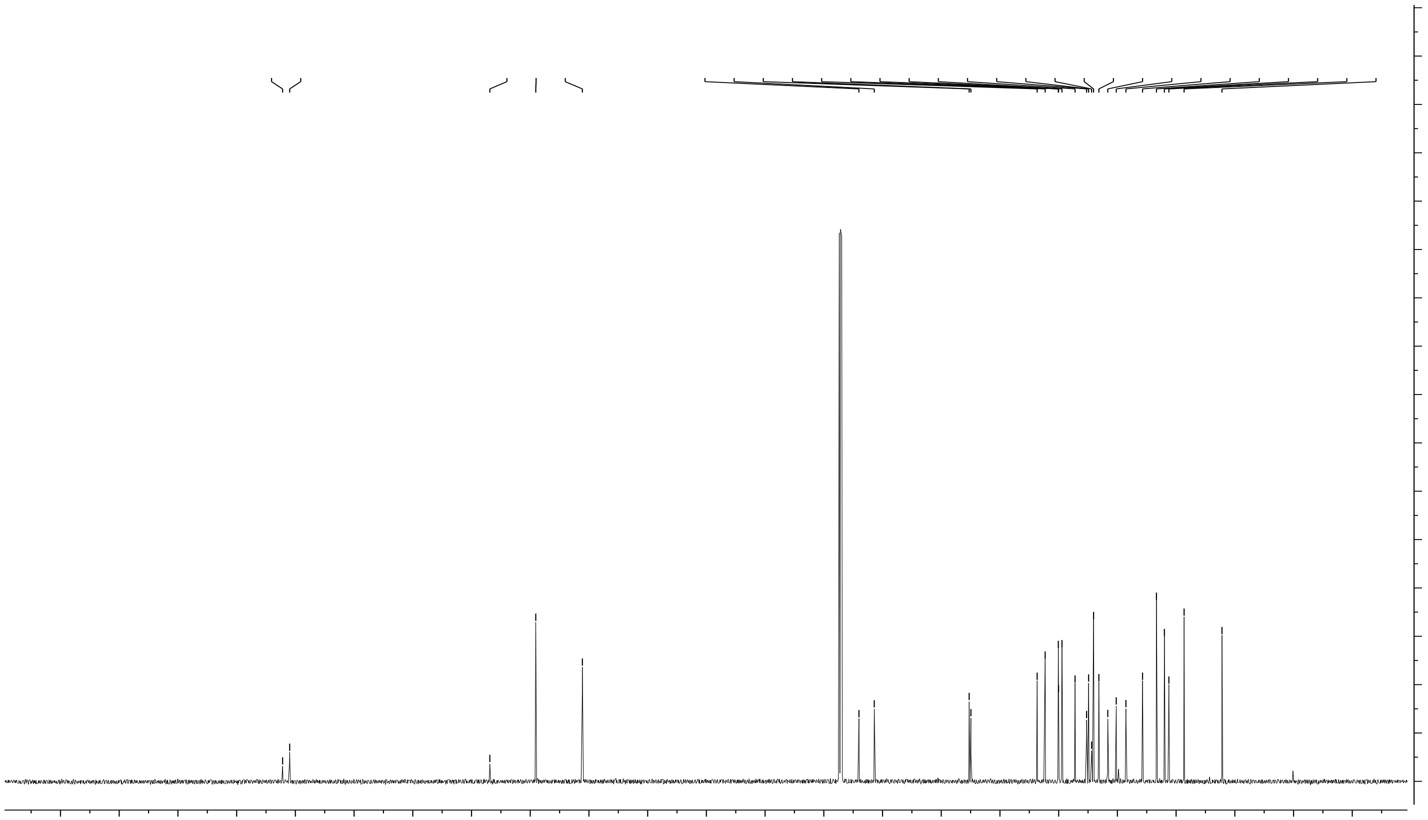

(3R,7S,10S,13R)-17-((R)-5-((4-chlorophenyl) amino)-5-oxopentan-2-yl)-3-h-ydroxy-10,13-dimethylhexadecahydro-1H-cyclopenta[a]phenanthren-7-yl acetate (MS) U12g:

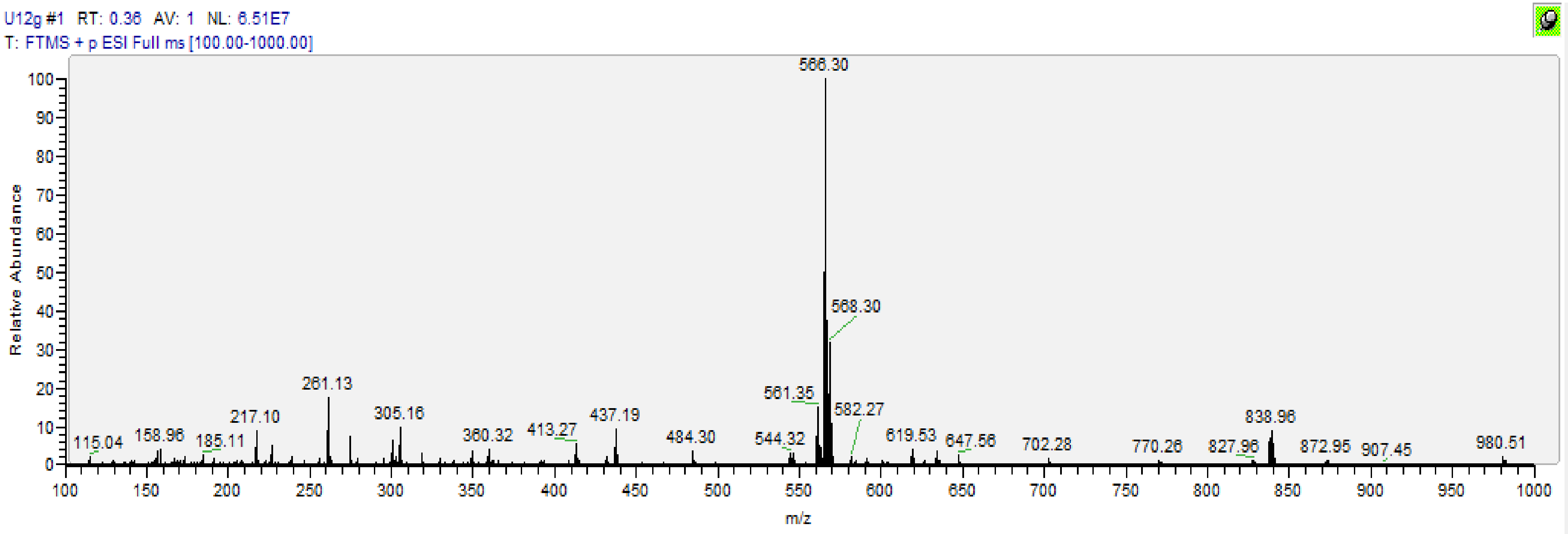

(3R,7S,10S,13R)-3-hydroxy-17-((R)-5-((4-hydroxyphenyl) amino)-5-oxopen-tan-2-yl)-10,13-dimethylhexadecahydro-1H-cyclopenta[a]phenanthren-7-yl acetate (^{1}H) U12h:

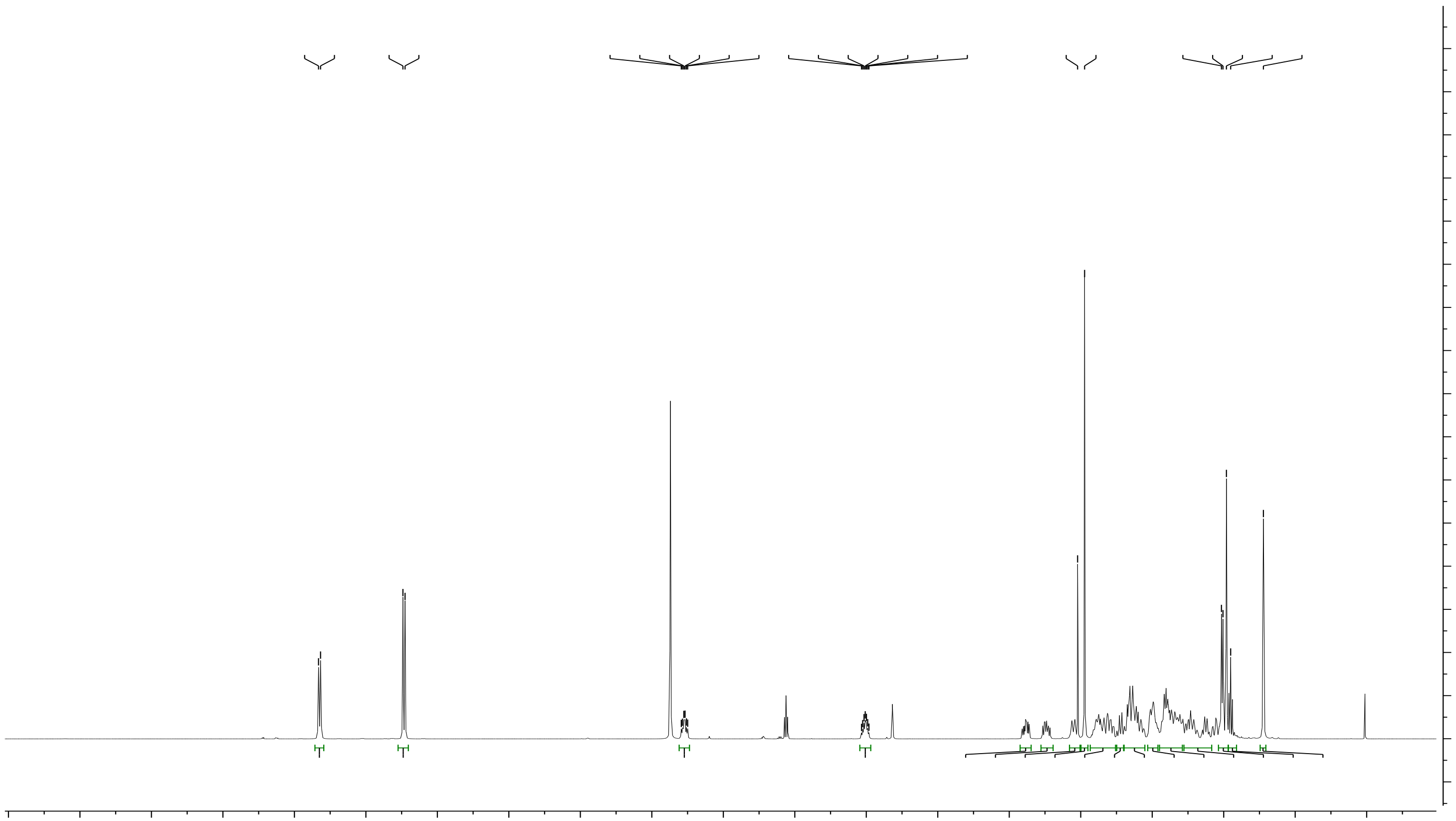

(3R,7S,10S,13R)-3-hydroxy-17-((R)-5-((4-hydroxyphenyl) amino)-5-oxopen-tan-2-yl)-10,13-dimethylhexadecahydro-1H-cyclopenta[a]phenanthren-7-yl acetate (^{13}C) U12h:

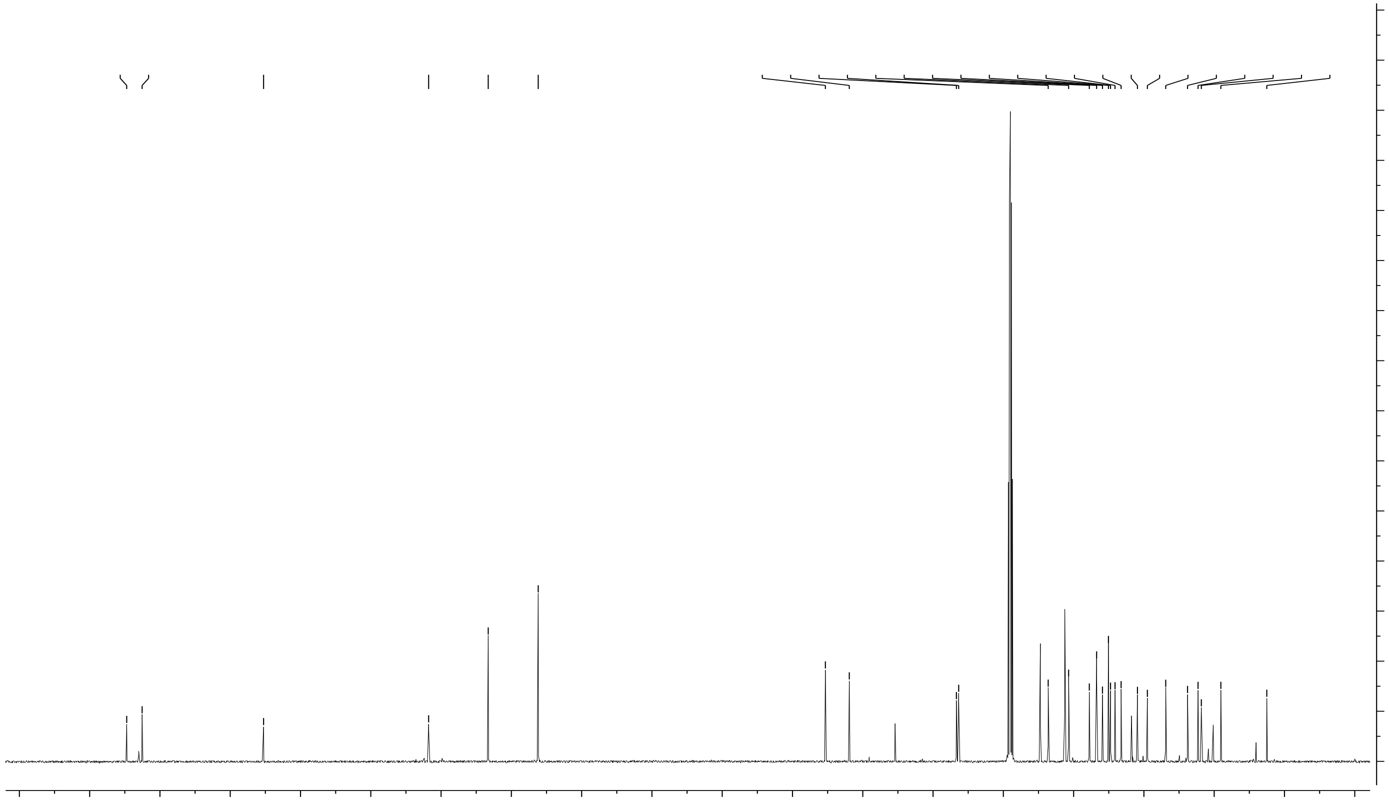

(3R,7S,10S,13R)-3-hydroxy-17-((R)-5-((4-hydroxyphenyl) amino)-5-oxopen-tan-2-yl)-10,13-dimethylhexadecahydro-1H-cyclopenta[a]phenanthren-7-yl acetate (MS) U12h:

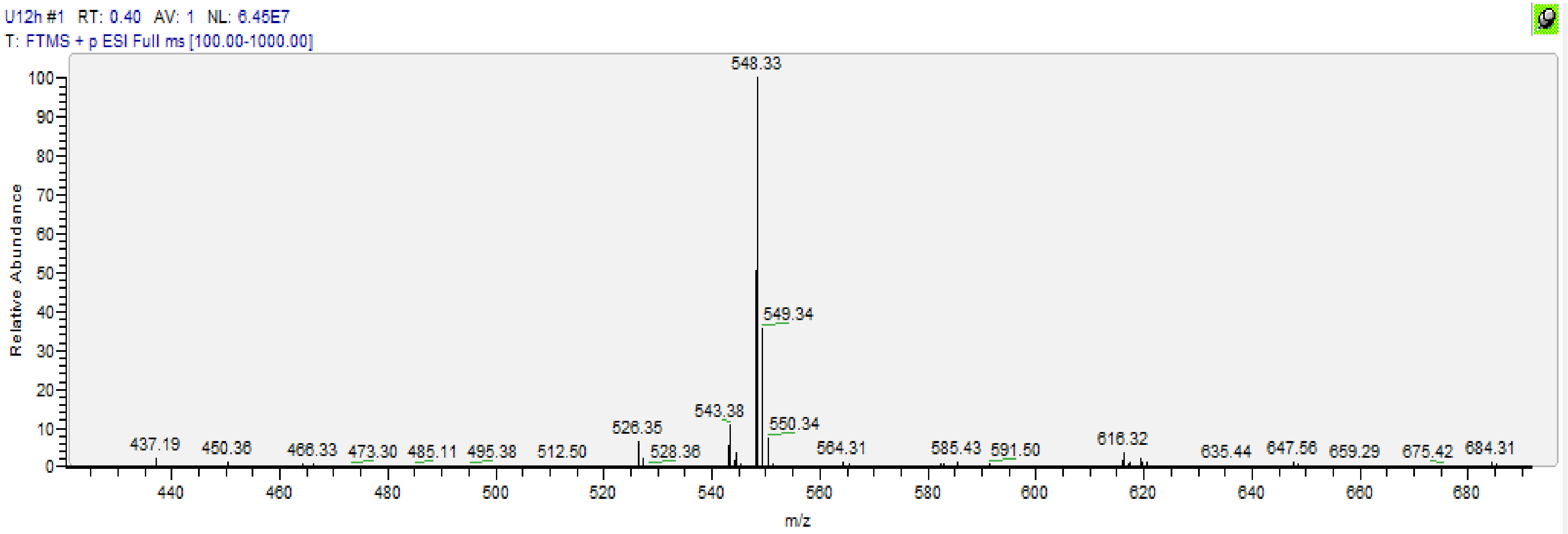

(3R,7S,10S,13R)-3-hydroxy-10,13-dimethyl-17-((R)-5-oxo-5-((4-(trifluoro-m-ethyl) phenyl) amino) pentan-2-yl) hexadecahydro-1H-cyclopenta[a]phenanthren-7-yl acetate (^{1}H) U12i:

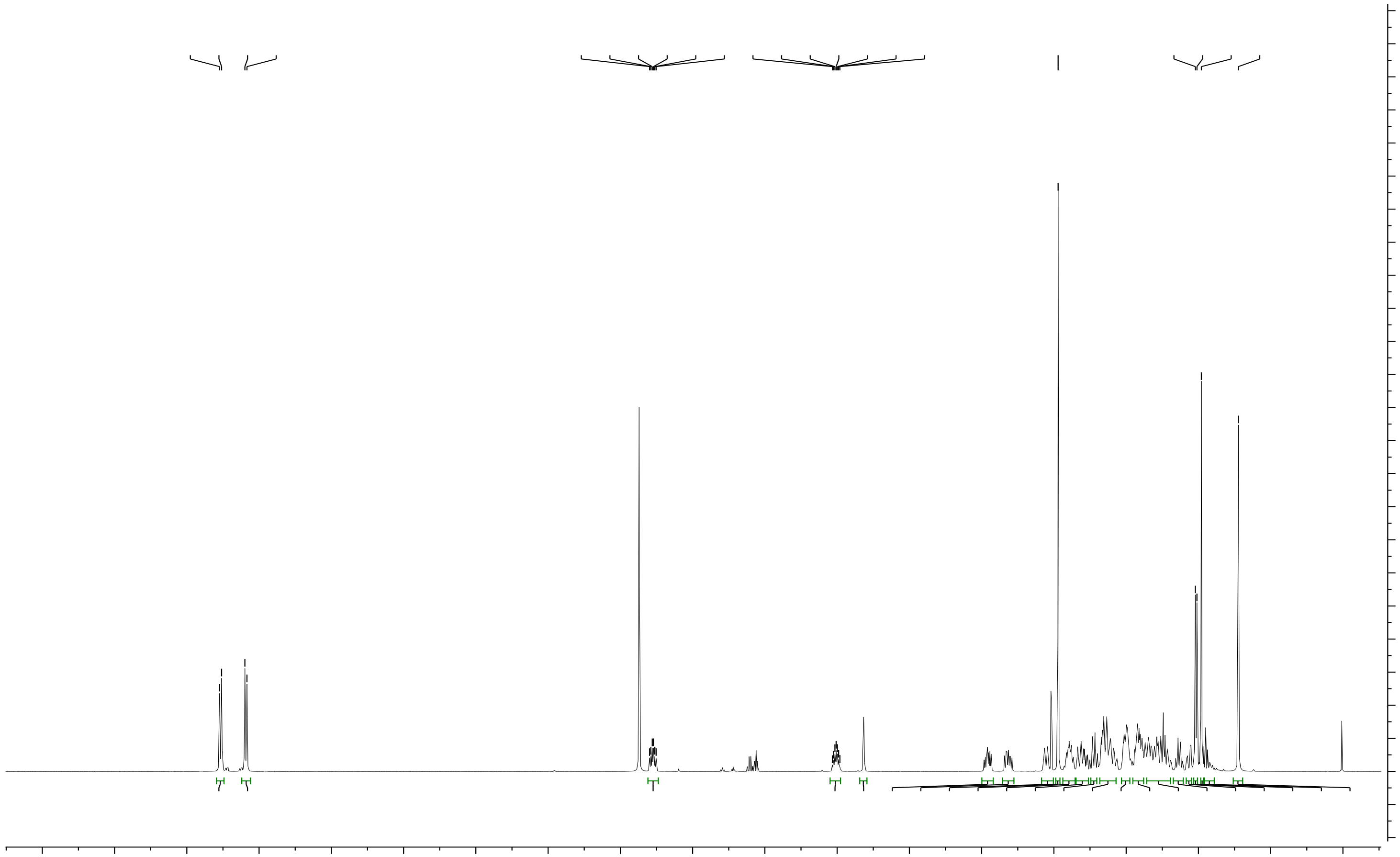

(3R,7S,10S,13R)-3-hydroxy-10,13-dimethyl-17-((R)-5-oxo-5-((4-(trifluoro-m-ethyl) phenyl) amino) pentan-2-yl) hexadecahydro-1H-cyclopenta[a]phenanthren-7-yl acetate (^{13}C) U12i:

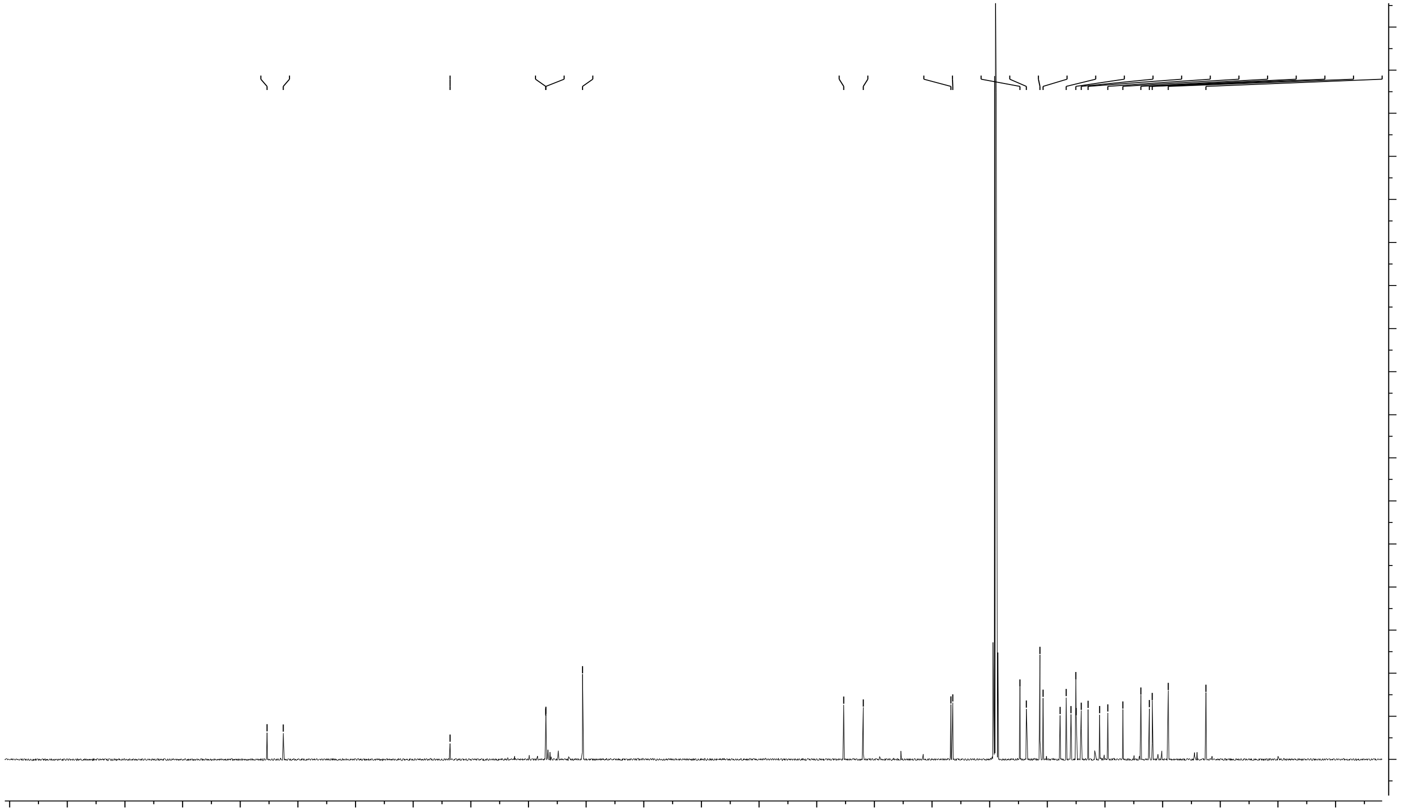

(3R,7S,10S,13R)-3-hydroxy-10,13-dimethyl-17-((R)-5-oxo-5-((4-(trifluoro-m-ethyl) phenyl) amino) pentan-2-yl) hexadecahydro-1H-cyclopenta[a]phenanthren-7-yl acetate (MS) U12i:

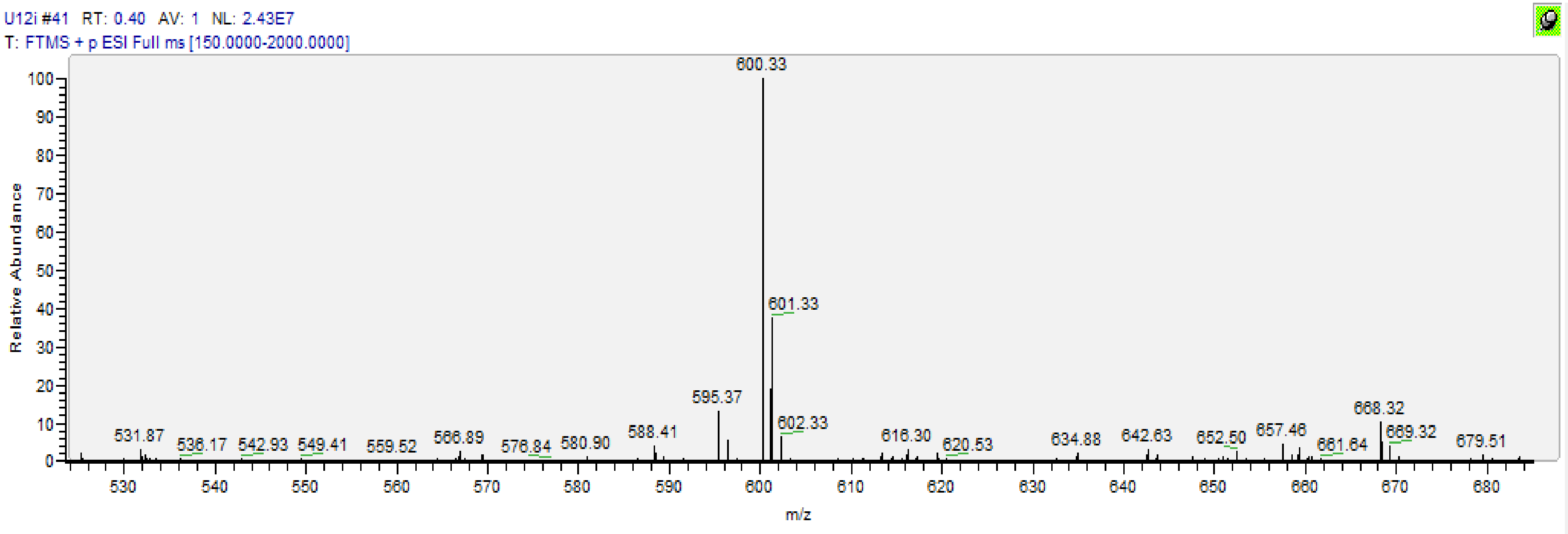

Supplement: Supplementary file 1 [file pharmaceuticals-15-00107-s001.zip › pharmaceuticals-1554665 supplementary(1).pdf]
